# Integrating multiplexing into confineable gene drives effectively overrides resistance in *Anopheles stephensi*

**DOI:** 10.1038/s41467-026-72835-5

**Published:** 2026-05-07

**Authors:** Mireia Larrosa-Godall, Lewis Shackleford, Matthew P. Edgington, Philip T. Leftwich, James C. Y. Luk, Joshua Southworth, Stewart Rosell, Jake T. Creasey, Jack M. Aked, Katherine Nevard, Alexander Dodds, Morgan Mckee, Eunice Adedeji, Estela Gonzalez, Joshua X. D. Ang, Michelle A. E. Anderson, Luke Alphey

**Affiliations:** 1 Department of Biology, University of York, Wentworth Way, York, UK; 2 York Biomedical Research Institute, University of York, Heslington, UK; 3 Arthropod Genetics, The Pirbright Institute, Pirbright, UK; 4 School of Biological Sciences, University of East Anglia, Norwich Research Park, Norwich, UK; 5 Present Address: The Francis Crick Institute, London, UK; 6 Present Address: Department of Mathematics and Statistics, University of Reading Whiteknights, Reading, UK; 7 Present Address: Animal and Plant Health Agency, Surrey, UK

**Keywords:** Population genetics, CRISPR-Cas9 genome editing, Genetic engineering

## Abstract

*Anopheles stephensi* is a major malaria vector mainly present in southern Asia and the Arabian Peninsula. Since 2012 it has invaded several countries of eastern Africa, stimulating urgent efforts to develop more efficient strategies for vector control such as CRISPR/Cas9-based homing gene drives. Target site resistance due to end-joining repair is a significant challenge to the deployment of these systems. The use of multiple sgRNAs has the potential to solve this issue. Here we perform experimental crosses to assess the homing and cutting efficiency of both classical (e.g. four adjacent sgRNAs all in one construct) and additive (e.g. separate constructs each expressing a single sgRNA) multiplexing strategies targeting the *cardinal* locus, in the presence and absence of a resistance allele. We find resistance alleles at one sgRNA target site can be mitigated by the presence of the additional sgRNAs with either strategy, and do not significantly reduce the homing efficiency for either strategy, validating their effectiveness. Further modelling using parameters derived from the strains generated indicates that while both strategies can overcome resistance allele formation, the fitness of the drive-carrying alleles is a critical factor in determining the overall performance and persistence of a split drive.

## Abstract (web summary)

*Anopheles stephensi* is a major malaria vector invading East Africa. Here, authors demonstrate that multiplexing sgRNAs in CRISPR gene drives effectively overcomes target site resistance while maintaining high homing efficiency, however fitness of strains is the key to long-term performance.

## Introduction

In 2023, there were approximately 263 million cases and 597,000 deaths due to malaria, of which 94% and 95%, respectively, occurred in Africa^1^. The primary vectors of this disease in the African continent are *Anopheles gambiae s.s., An. coluzzii, An. arabiensis* and *An. funestus;* all of which have life cycles adapted to rural settings, resulting in malaria being an overwhelmingly rural disease^2^. In contrast, *An. stephensi*, the main malaria vector in India and Pakistan, is better adapted to urban environments. Its unique ability to locate clean water, usually in storage tanks, to lay its eggs^2^ instead of more typical larval breeding sites used by African native vectors such as puddles and ditches, allows it to avoid polluted water (e.g. oil and sewage) in urban areas^3^. This adaptation allowed *An. stephensi* to thrive in urban areas, driving malaria outbreaks in cities across India, Pakistan, the Arabian Peninsula and Iran^4^. Since the first reported finding in Djibouti in 2012, *An. stephensi* has invaded west through Africa, expanding its range into Ethiopia and Sudan in 2016, Somalia in 2019, Nigeria in 2020, Kenya and Ghana in 2022, and recently in the Republic of Niger (2025)^5–9^. This threatens the 40% of sub-Saharan Africans living in urban areas and puts an additional 126 million people at risk of malaria^10^. Vector control interventions such as indoor residual spraying (IRS) and insecticide-treated nets (ITNs) are currently used to control malaria transmission. However, the efficiency of these strategies has been threatened by an increase in insecticide resistance by mosquitoes^7,11^. As a result, in 2023, the WHO released an initiative to stop the spread of this species in Africa, calling for prioritising research to evaluate new tools to control *An. stephensi*^11^.

The ability to modify DNA using recently available tools such as clustered regulatory interspaced short palindromic repeats- associated protein 9 (CRISPR/Cas9) has given rise to alternative approaches that use genetic manipulation to reduce vector competence or suppress wild mosquito populations with relatively small initial releases^12,13^. The Cas9 endonuclease targets a specific sequence determined by the single guide RNA (sgRNA) and initiates a double-stranded break. The DNA is repaired by cellular mechanisms such as homology-directed repair (HDR) or non-homologous end joining (NHEJ). By using CRISPR/Cas9 to bias the inheritance of a cargo gene through HDR, populations of mosquitoes could be modified to be refractory to malaria infection^14^ or, by targeting a gene vital for fertility, they could suppress populations^15^. For both strategies, repair through the NHEJ pathway will often result in a change to the sequence of the target site, generating an allele which is resistant to further sgRNA recognition and cleavage by Cas9; individuals which inherit these mutations are also immune to future drive allele conversion^16–20^. These cut-resistant mutants can be functional (*r*1) or non-functional (*r*2) and pose a threat to a gene drive’s ability to spread and persist in a population^21^. Such formation of cut-resistant alleles is more likely to occur when embryos contain maternally deposited Cas9 which may produce cuts early in development^16^. High rates of cut-resistant allele formation can threaten the success of any form of gene-drive; if the drive has a significant fitness cost, even relatively low rates may be problematic if the cut-resistant allele then has a significant fitness advantage over the drive-bearing allele^16,22,23^.

One promising strategy to mitigate the threat of resistance alleles is to multiplex, i.e. designing a drive construct which expresses multiple sgRNAs targeting the same gene^20,24–28^. In the classical multiplexing strategy, a single construct expresses multiple sgRNAs, which cut adjacent target sites and resistance allele formation at one or even multiple target sites would not halt the spread of a gene drive into the population as long as there is at least one remaining intact target site. In theory, this increases the number of subsequent NHEJ events required to form a fully drive-resistant allele. This also decreases the probability and frequency of completely drive-resistant individuals generated in a population. One concern when utilizing some multiplexing strategies that require a deletion of some sequence surrounding the sgRNA target sites is the effect of sequence heterology or dissimilarity between the homology arms of the donor template and the cut chromosome on drive efficiency, due to the requirement to resect the cut allele some distance to reach homologous sequences^27–29^. CRISPR/Cas9-based gene drives in Anopheline mosquitoes have been shown to be tolerant of such sequence heterology, with no reduction in homing with up to 6.6% target locus heterology^30,31^. Multiplexing with multiple sgRNAs can also reduce the overall likelihood of accumulated mutations being functional, as each target site is cut and repaired, giving additional opportunities for the generation of r2 alleles at each target. A significant drawback to the classical multiplexing strategy is that if the two outermost targets sites are cut simultaneously, the entire target region between them could be deleted either by NHEJ or microhomology-mediated end joining (MMEJ), removing all target sites in one step, though with careful design this is unlikely to result in a functional protein (r1)^20,23,32–34^. Therefore, alternative multiplexing strategies that allow multiplexing at the population level instead of the individual level have been proposed and their theoretical properties explored using mathematical modelling^24^. These strategies can target single or multiple genes through multiple, distinct constructs expressed in different individuals, thereby multiplexing on a population level, as opposed to the classical strategy where multiple adjacent sites are targeted by a single construct, multiplexing at the individual level. Such strategies could be used sequentially in populations such that if resistance alleles accumulate to levels that impede a given drive, individuals carrying the alternative element could then be released. These would be unaffected by the pre-existing resistance alleles to the initial drive. One of the proposed strategies, the additive strategy, is characterised by targeting different sites spaced so they are unlikely to segregate from each other through recombination but also are distant enough that homing at each target site can occur independently, usually within the same gene. Modelling of the additive strategy has shown that its use increases the number of generations in which gene drives persist at high frequencies compared to the classical multiplexing strategy, but this has yet to be empirically explored^24^. While the focus here is on cut-resistant mutations induced by end-joining, the advantages of multiplexing apply equally to standing genetic variation, i.e. naturally occurring SNPs.

Genes in the ommochrome pathway have often been used as targets for homing-based gene drives in Anopheline mosquitoes. Genetic manipulation of this pathway affects eye colour, making the gene involved experimentally tractable with homozygous nulls presenting a distinctive eye phenotype; null mutations of their homologues in *Drosophila melanogaster* are viable and fertile. Previous studies have targeted two genes in this pathway; *kynurenine 3-monooxygenase* (*kmo*), which resulted in a reduction in the survival after blood-feeding in homozygous *An. stephensi* females^16,35^, and *cardinal* (*cd*), which did not show any apparent reproductive or survival fitness cost in *An. gambiae* mosquitoes^14^. Based on this information *cd* was selected as a putative neutral target gene for this study in *An. stephensi*, and a potential target locus for a population modification drive. Here, we empirically investigate multiplexing strategies proposed in previous theoretical work to mitigate the risk of resistance allele formation targeting the *cd* gene. We demonstrate the ability of two strategies, the classical and additive, to continue homing in the presence of a resistance allele. However, we found that, likely due to high cleavage rates, in the rare instances when homing did not occur, the classical multiplexing design frequently generated large deletions which removed several sgRNA target sites simultaneously. Modelling indicates that this type of deletion is the most frequent cause for the classical multiplexing design to fail^24^. We demonstrate through modelling that the additive strategy can successfully bypass this issue. However, this strategy is sensitive to the fitness costs of successive insertions.

## Results

### The classical multiplexing strategy gives high homing rates despite the requirement for resection

**Fig. 1:**
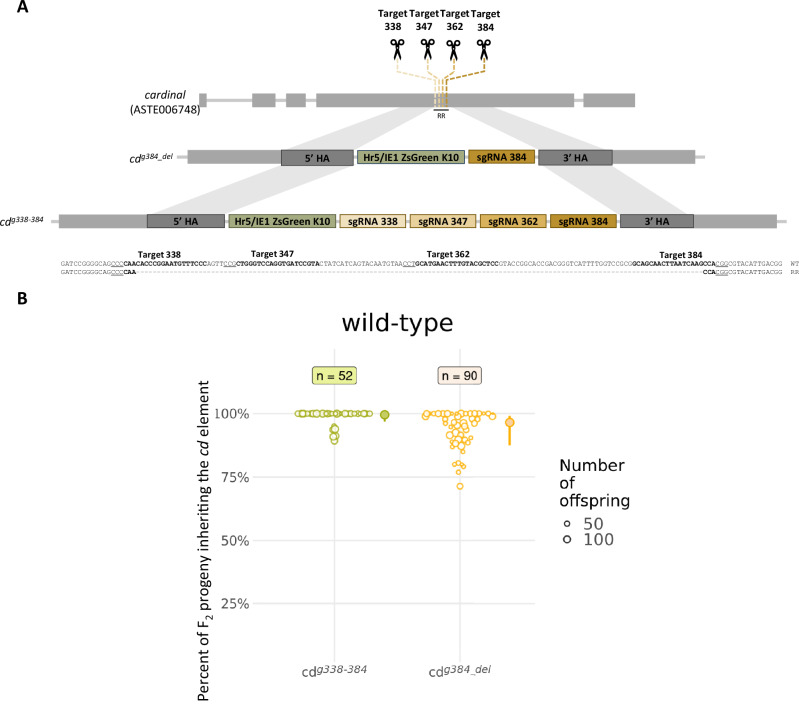
Gene drives using the classical multiplexing strategy have high efficiency. **A** Schematic representation of the *cd* transcript in *An. stephensi*, and the *cd*^*g384_del*^ and *cd*^*g338-384*^ HDR knock-in constructs with their corresponding insertion sites. **B** Inheritance rates of the *cd*^*g384_del*^ and *cd*^*g338-384*^ transgenes from trans-heterozygotes with *zpg*^*3’Cas9*^. Circles represent the percentage of F_2_ progeny which inherited the *cd*^*g338-384*^ (green) or the *cd*^*g384_del*^ (yellow) transgenes and the size of the circle is proportional to the number of progeny obtained from each female. Filled circles and error bars represent the mean and the 95% CI, respectively, calculated from a generalized linear mixed model, with a binomial (‘logit’ link) error distribution. Full statistical reporting is provided in text. WT wild-type; HA homology arm; RR resected region; *n* = number of female individuals whose progeny were scored. Source data are provided.

We previously have used a split-drive targeting the *cd* gene and shown >98% inheritance using a transgenic line inserted into *An. stephensi cd*, in the 384 sgRNA target site expressing a single sgRNA from the *As*7SK promoter (*cd*^7SK^, here referred to as *cd*^*g*384^ to distinguish from other *cd* targets) and a line expressing Cas9 from the endogenous *zpg* locus (*zpg*^*3’Cas9*^)^36^. To analyse the feasibility of the classic multiplexing strategy to mitigate target site resistance, a construct carrying a *Hr5/IE1-ZsGreen-K10* fluorescent marker and four sgRNAs expressed by four different RNA pol III promoters^36^, with four adjacent targets in *cd*, was designed for HDR insertion into this same target region of the *cd* gene (Fig. 1A). The target sites of all four sgRNAs are within 136 nucleotides (nt), aiming to preserve homing efficiency while reducing the likelihood of the creation of functional mutations. The resulting plasmid was used to generate the *cd*^*g*338-384^ line through CRISPR/Cas9-mediated integration into *cd* (Fig. 1A). A second construct and transgenic line containing the same homology arms (keeping the 136nt deletion of the additional sgRNA target sites) and marker cassette as the *cd*^*g338-384*^ line but only expressing one sgRNA recognizing the 384 cut site was also generated, *cd*^*g384_del*^ (Fig. 1A). This line serves to determine the effect of resection on drive conversion efficiency.

To determine drive efficiency we first combined the Cas9-expressing transgenic line with the sgRNA-expressing line by separately crossing *cd*^*g384_del*^ or *cd*^*g338-384*^ heterozygous males to heterozygous *zpg*^*3’Cas9*^ females to obtain trans-heterozygotes expressing both sgRNA and Cas9 (Supplementary Data 1). We could then measure homing and cutting in the germline of these Cas9 and sgRNA-expressing mosquitoes by crossing them to suitable genotypes and observing their progeny. Crosses to wild type would allow measurement of homing efficiency, indels generated by NHEJ are indistinguishable from wild type alleles in these progeny due to the recessive phenotype of *cd* mutants. To be able to recognise NHEJ-generated mutant alleles we crossed trans-heterozygotes to a previously established *cd* knockout line (*cd*^*384R*^, a 3 bp deletion in *cd* which gives a pink-eyed phenotype when homozygous). This still allows observation of homing by measuring the inheritance of the *cd* insertion as above, but we can also observe NHEJ mutations because the homologous chromosome carries a null allele, which results in a clearly visible pink-eyed phenotype. We therefore crossed trans-heterozygous males to females, which were homozygous mutants for *cd* (*cd*^*384R*^) and the offspring of each female were separately screened for the presence of the fluorescent markers (inheritance above 50% indicating homing) as well as for eye phenotype (indicating mutation in *cd*, which indicates cutting followed by homing or end-joining, Supplementary Data 1). The inheritance rate of *cd*^*g384_del*^ (96.48%; [95% CI] = [87.49–99.08]) (Fig. 1B, Supplementary Table 1) was significantly lower (OR = 0.16; *z *= −2.13; *p* = 0.033, Supplementary Data 2) than the inheritance of *cd*^*g384*^ (99.43% [98.41–99.8]), which expresses the same sgRNA but from within homology arms which perfectly match the sgRNA cut site^36^. This indicates that the requirement for resection during repair reduces the inheritance rate at this target, here by about 3%. However, this slight reduction in the inheritance rate was reversed by including the three additional sgRNAs in the multiplexed version, *cd*^*g338-384*^. Using the same series of crosses (Supplementary Data 1) we found the *cd*^*g338–384*^ transgene was inherited by 99.55% ([96.87–99.94]) of the progeny (Fig. 1B, Supplementary Table 1), which was not significantly different from the inheritance bias observed for the *cd*^*g384*^ line (OR = 1.26; *z* = 0.21; *p* = 0.836, Supplementary Data 2). No statistically significant differences were observed between the inheritance bias of *cd*^*g384_del*^ and *cd*^*g338-384*^ (OR = 0.12, *z* = 1.72, *p* = 0.09, Supplementary Data 2). We separately reviewed cutting rates, as the frequency of observed mosaicism in the eye colour phenotype (Supplementary Data 1), this was observed at 100% for the *cd*^*g338–384*^ transgene (and estimated at >99.9% [99.8–99.9] with a robust glm model), with modest decreases in cleavage rates for the *cd*^*g384*^ line (99.8% [99.7–99.9]; *z* = −1.99; *p* = 0.04) and the *cd*^*g384_del*^ line (98.3% [97.8–98.8]; *z* = −3.48; *p* < 0.001).

### Inheritance bias is caused by homing and not meiotic drive

To verify that the inheritance bias observed for the *cd*^*g384*^ (data published in ref. ^36^) and the *cd*^*g338-384*^ lines was due to homing and not meiotic drive, we crossed *zpg*^*3’Cas9*^ heterozygous females to AGG1928 heterozygous males (F_0_) to obtain *zpg*^*3’Cas9*^*; AGG1928* trans-heterozygotes (F_1_). The AGG1928 transgene, which was generated by *piggyBac*-mediated random integration, was found to be linked to the *cd* gene (less than 1% recombination rate) and contains a *DmAct5C-ZsYellow-P10* marker cassette as well as other components not relevant for this study. In this experiment, *zpg*^*3’Cas9*^*; AGG1928* trans-heterozygous males were crossed to *cd*^*g384*^ or *cd*^*g338-384*^ females, and the remaining crosses were performed the same as described above (Supplementary Data 1). If meiotic drive occurs we would expect to see a decrease in the inheritance of the marked allele from the expected Mendelian inheritance rate of 50% (Supplementary Fig. 1). Despite an inheritance rate of 93.3% ([95% CI] = [92.1–94.2]) for *cd*^*g384*^ and 97.4%([95% CI] = [96.6–98.1]) for *cd*^*g338-384*^, we found no evidence for meiotic drive in the inheritance rate of AGG1928 in either *cd*^*g338-384*^ (49.6%; [95%CI] = [47.2–52]) or *cd*^*g384*^ (48.3%; [46.2–50.5]) (GLMM LRT: *χ*^2^_1_ = 0.61, *p* = 0.434; Supplementary Table 2) crosses.

### Classical multiplexing gives high homing in the presence of a resistance allele

**Fig. 2:**
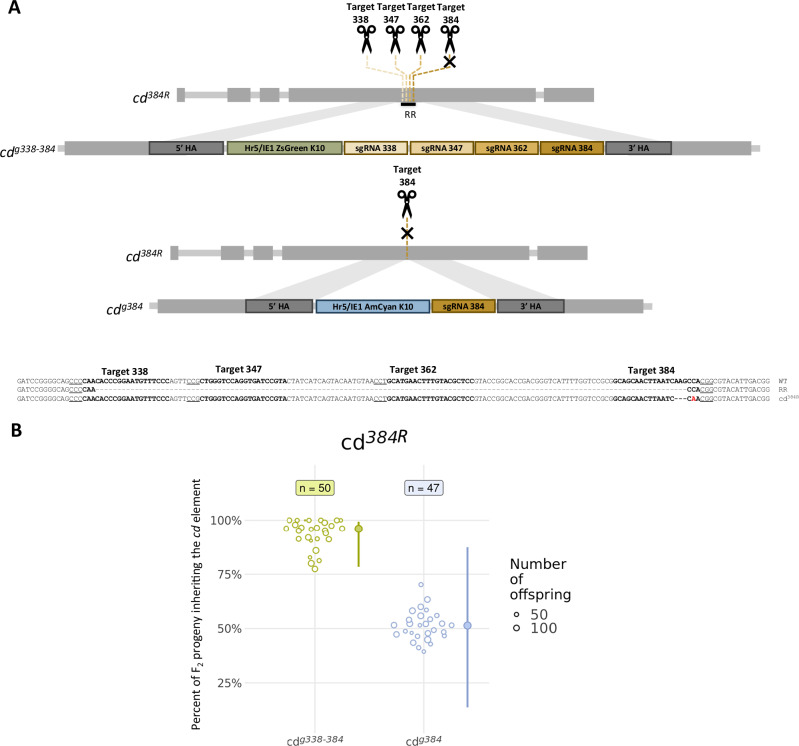
Classical multiplexing can sustain high inheritance rates even in the presence of resistance. Schematic representation of the *cd* transcript in *An. stephensi* including the *cd*^*g338-384*^ and *cd*^*g384*^ HDR knock-in constructs with their corresponding insertion sites (**A**). The black cross represents the location of the mutation at the 384 cut site. Sequence alignment to illustrate the resected region (RR), and the sequence of the *cd*^*384R*^ mutation. **B** Circles represent the percentage of F_2_ progeny which inherited *cd*^*g384*^ (blue) and *cd*^*g338-384*^ (green) from trans-heterozygotes with *zpg*^*3’Cas9*^ in the background of a homozygous mutation at the 384 cut site. The size of the circle is proportional to the number of progeny obtained from each female. Filled circles and error bars represent the mean and the 95% CI, respectively, calculated from a generalized linear mixed model, with a binomial (‘logit’ link) error distribution. Full statistical reporting is provided in text. WT wild-type, RR resected region, HA homology arm, *n* = number of female individuals whose progeny were scored. Source data are provided.

To assess the ability of the classic multiplexing strategy to surpass resistance we generated a Cas9-expressing line, which was also homozygous for the previously utilised 3 bp deletion in the sgRNA target site in *cd* (*zpg*^*3’Cas9*^*;cd*^384R^). In this strain, the sgRNA 384 target site can no longer be cleaved due to the change in the nucleotide sequence, so further homing relies on the other three sgRNAs. Those *zpg*^*3’Cas9*^*;cd*^*384R*^ females were crossed to *cd*^*g338-384*^ heterozygous males (F_0_) to obtain *zpg*^*3’Cas9*^*;cd*^*g338-384/384R*^ trans-heterozygous males (Fig. 2A, Supplementary Data 1), which were then crossed to *cd*^*384R*^ females (F_1_). The F_2_ progeny were screened for fluorescence. We found 96.07% ([95% CI] = [78.43–99.39]) of the F_2_ progeny inherited the *cd*^*g338-384*^ allele (Fig. 2B, Supplementary Table 1). Even though the observed inheritance bias was slightly lower than the inheritance observed in the absence of resistance (99.55% ([95% CI] = [96.87–99.94]), this was not statistically significant (OR = 0.11, *z* = 1.58, *p* = 0.11, Supplementary Data 2), showing that the classic multiplexing strategy can sustain high drive efficiency in the presence of a cut-resistant allele. A cross was also performed following the same scheme (Supplementary Data 1) but using the singleplex *cd*^*g384*^ line (Fig. 2A). The *cd*^*g384*^ transgene was inherited at a Mendelian rate (51.38% [95% CI] = [13.81–87.45]), consistent with the hypothesis that the 3 bp deletion would prevent recognition and cutting by the only sgRNA present in that transgene (Fig. 2B).

### The additive multiplexing strategy maintains homing in the presence of a resistance allele

The additive multiplexing strategy for population-level multiplexing relies on a series of single sgRNA expressing constructs targeting sites which are close enough together that recombination events between them should be rare, but far enough apart that cleaving and homing of one would not affect the other^24^. In practical terms, these targets can target the same gene separated by a few hundred nucleotides. Although modeling in our previous work^24^ suggested that this strategy would be less effective than the classical multiplexing approach, we selected it for experimental testing because its development was more straightforward, the necessary lines were already available in the laboratory, and we anticipated that analysis would provide general lessons.

**Fig. 3:**
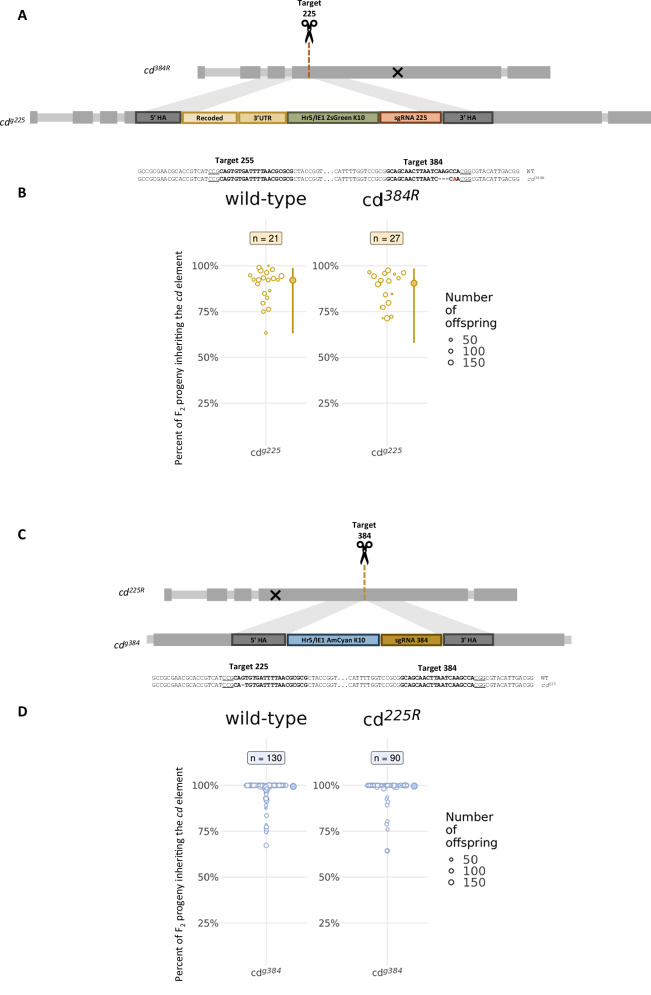
Additive multiplexing maintains high inheritance rates in the presence of pre-existing mutant alleles. Schematic representation of the *cd*^*g225*^ (**A**) and the *cd*^*g384*^ (**C**) transgenes and their insertion sites located within the *cd* transcript and sequence alignment of the *cd* mutants. The dashed line with the scissors represents the cut site and the black x indicates the location of the homozygous resistant mutation at either target site 384 or 225 in the *cd* gene in the background of *zpg*^*3’Cas9*^. **B** Inheritance rates of *cd*^*g225*^ from trans-heterozygotes with *zpg*^*3’Cas9*^ in a wild-type background (left) or in the background of a resistance allele (right). **D** Inheritance rates for the *cd*^*g384*^ allele from trans-heterozygotes with *zpg*^*3’Cas9*^ in a background with (right) or without (left) a deletion affecting the contiguous target site. Circles represent the percentage of F_2_ offspring that inherited the *cd*^*g225*^ (**B**) or the *cd*^*g384*^ transgene from each female (**D**). Data for *cd*^*g384*^ in a wild-type background presented here was originally published in ref. ^36^. Filled circles and error bars represent the mean and 95% CI calculated from a generalized linear mixed model, with a binomial (“logit” link) error distribution. Full statistical reporting is provided in text. WT: wild-type; HA: homology arm; *n* = number of female individuals whose progeny were scored. The size of the circle is proportional to the number of progeny obtained from the female. Source data are provided.

To validate the additive multiplexing strategy, we generated a transgenic line composed of an sgRNA-expressing cassette targeting and inserted into a site upstream of the 384 target site. For this strategy to be successful the distance between the target sites must be such that both transgenes can home independently while reducing the probability of segregation via recombination. Therefore, we selected a second target 475 nucleotides away from the 384 target, *cd*^*g225*^ (Fig. 3A). The *cd*^*g225*^ line was then crossed to *zpg*^*3’Cas9*^ (F_0_) to determine the homing and cutting rates at the 225 target site (Supplementary Data 1). We found 92.08% ([95% CI] = [63.12–98.75]) of the F_2_ progeny inherited the *cd*^*g225*^ allele, indicating that the inheritance rate (OR = 0.07; *z* = −2.44; *p* = 0.015) and cleavage rate (95.8% [95.01–96.43]; z = −14.57; *p* < 0.001, Supplementary Data 2) at the 225 cut site, while high, are significantly lower than the rate obtained at the 384 site (Fig. 3B, Supplementary Table 1).

We also wanted to assess the homing and cutting rates of *cd*^*g225*^ or *cd*^*g384*^ in the presence of a resistant allele at the distant target site (Fig. 3A and C, Supplementary Table 1) to determine the behaviour of a drive in a population with resistance alleles to a previously released gene drive. For this purpose, we generated an additional strain of the *zpg*^*3’Cas9*^ line to be homozygous for a resistance allele at the 225 sgRNA target site (*zpg*^*3’Cas9*^;*cd*^*225R*^). Female *zpg*^*3’Cas9*^*;cd*^*384R/384R*^ were crossed to *cd*^*g225*^ males and *zpg*^*3’Cas9*^;*cd*^*225R/225R*^ females were crossed to *cd*^*g384*^ males (F_0_) to obtain trans-heterozygotes. Then, trans-heterozygous males for the *zpg*^*3’Cas9*^*;cd*^*384R*^ and *cd*^*g225*^ alleles and for the *zpg*^*3’Cas9*^;*cd*^*225R*^ and *cd*^*g384*^ alleles were separately crossed to *cd*^*384R*^ and *cd*^*225R*^ females (F_1_), respectively. F_2_ offspring were screened for fluorescence expression (Supplementary Data 1). The *cd*^*g225*^ transgene was inherited by 90.5% ([95%] = [57.88–98.51]) of the F_2_ offspring (Fig. 3B, Supplementary Table 1), which was slightly lower but not significantly different from the inheritance rates in the absence of resistance at the distal sgRNA target site (OR = 1.22; *z* = 0.14; *p* = 0.89, Supplementary Data 2). Similarly, the *cd*^*g384*^ allele was inherited by 99.59% ([95% CI] = [98.31–99.9]) of the F_2_ progeny (Fig. 3D, Supplementary Table 1). These results suggest that multiple releases of independent single-target CRISPR/Cas9-based gene drives could spread in a population in the presence of resistance.

### Deep sequencing reveals variable sgRNA efficiency and generation of resistance alleles

**Fig. 4:**
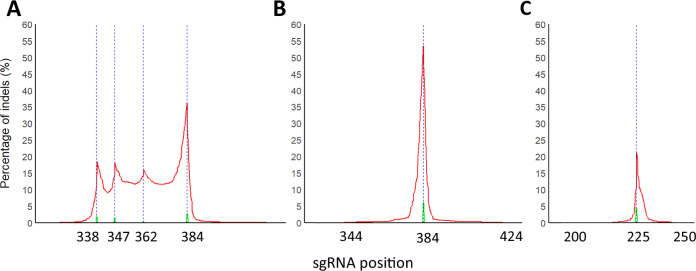
Rates of insertions and deletions (indels) in the target sites of *cd*^*g338-384*^, *cd*^*g384*^ and *cd*^*g225*^. Blue dashed lines represent the cut sites of the different sgRNAs. Red line represents the rate of deletions while green line represents the rate of insertions in the *zpg*^*3’Cas9*^*;cd*^*g338-384*^ (**A**), the *zpg*^*3’Cas9*^;*cd*^*g384*^ (**B**), and the *zpg*^*3’Cas9*^;*cd*^*g225*^ (**C**) F_1_ trans-heterozygous males determined by CRISPResso2. Source data are provided.

To determine the spectrum of potential resistance alleles generated by the *cd*^*g338-384*^ multiplexed line, we collected F_1_ *zpg*^*3’Cas9*^*;cd*^*g338-384*^, *zpg*^*3’Cas9*^;*cd*^*g384*^ and *zpg*^*3’Cas9*^;*cd*^*g225*^ trans-heterozygous males for Illumina-based amplicon sequencing of the *cd* target sequence and analysis using CRISPResso2^37^. The sgRNA384 expressed by *zpg*^*3’Cas9*^*;cd*^*g384*^ trans-heterozygous males had the highest mutation rate of all the sgRNAs analyzed (63%, Fig. 4B). This sgRNA also showed the highest mutation rate (46.1%) in the *zpg*^*3’Cas9*^*;cd*^*g338-384*^ F_1_ trans-heterozygous males while the other three sgRNAs showed relatively lower mutation rates (22.5–26%) at their respective target sites (Fig. 4A). sgRNA225 had one of the lowest overall indel rates (26%, Fig. 4C). Analysis of wild-type males showed that all nucleotides within the analysis window were 95.5% identical to the reference sequence, with the remaining reads mostly modified by single nucleotide substitutions (SNPs, 97.7% of the modified reads, Supplementary Fig. 2), which we exclude from our analysis. Manual analysis of sequences with indels revealed approximately 75% of indels were out-of-frame in all the transgenic lines (Supplementary Table 3), indicating that although drive resistant alleles are generated, they are likely to be non-functional. The *zpg*^*3’Cas9*^;*cd*^*g384*^ had the highest proportion of out-of-frame indels (78.5%), compared with that observed in the *zpg*^*3’Cas9*^*;cd*^*g338-384*^ [70.1%] and *zpg*^*3’Cas9*^;*cd*^*g225*^ (71.7%).

**Fig. 5:**
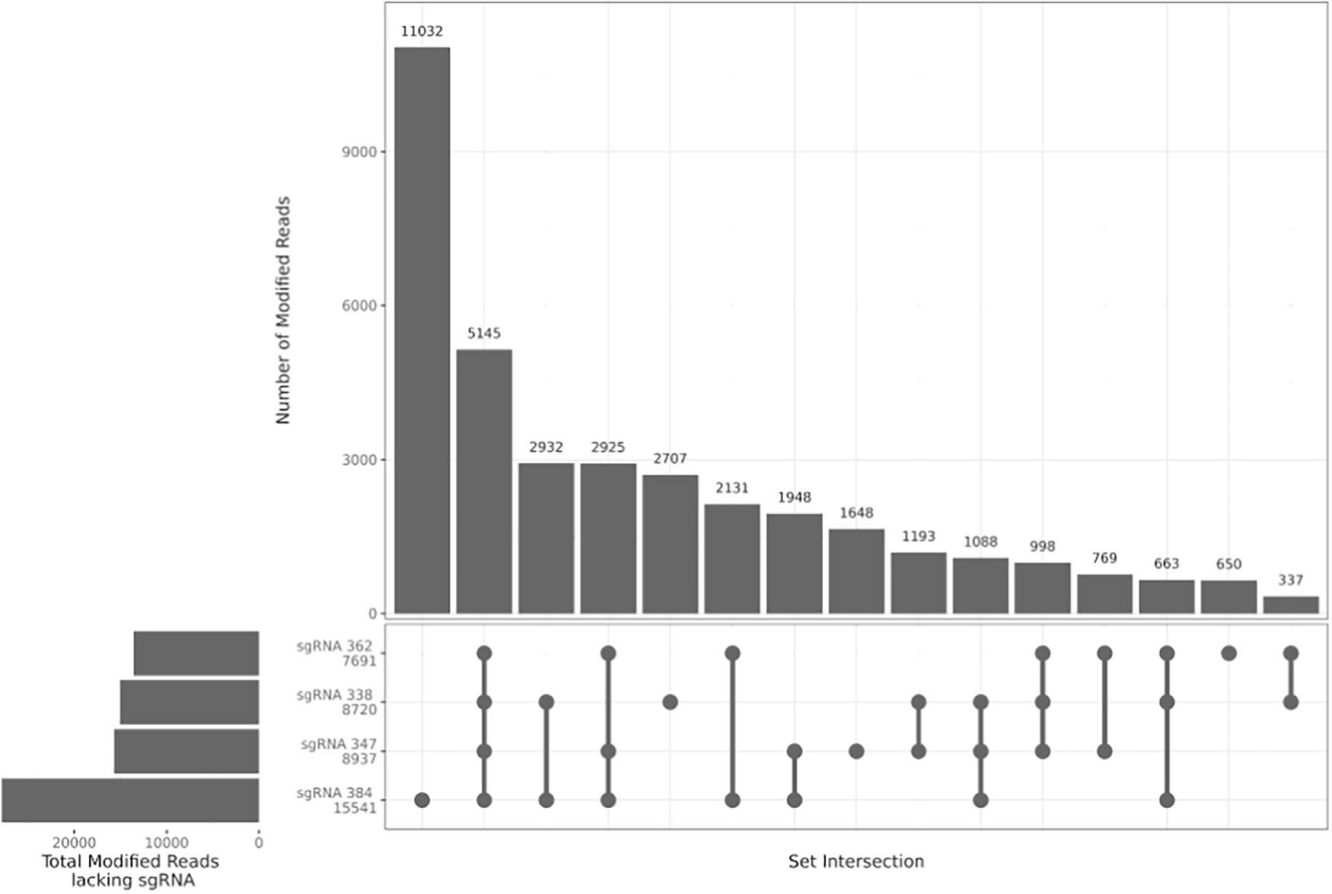
UpSet plot showing the number of modified reads lacking each sgRNA target site, including all possible combinations. The number of modified reads for each combination of targets is indicated above each column. The number under the sgRNA name is the number of unmodified reads for that target site. Source data is provided.

Further analysis of the reads in *zpg*^*3’Cas9*^*;cd*^*g338-384*^ trans-heterozygotes reveals 18% of total reads (30% of modified reads) lack just the sgRNA384 target site and 8.5% of total reads (14% of modified reads) lack all four sgRNA target sites (Fig. 5, Supplementary Table 4). Of the reads lacking all four target sites, 68% had a single deletion which ablated the target sites. This suggests that simultaneous cutting of the two outermost sgRNAs, 338 and 384, can directly eliminate all four sgRNA targets and create a fully cut-resistant allele in a single step.

Of the ten most common mutations, two have a large deletion that removes all four sgRNA targets, another two have deletions which remove three targets and the remaining six have mutations only at the 384 target site (Supplementary Fig. 3).

### Modelling drive behaviour

The above work demonstrated the potential of genetic elements for both classical and additive multiplexing approaches to achieve high levels of sgRNA target site cleavage and gene drive inheritance. Thus, we use parameters derived here to inform mathematical models, allowing us to further explore the potential ability of these approaches to overcome issues of gene drive resistance and fitness costs alongside their ability to provide lasting population modification.

Mathematical models used here are analogous to those previously used to compare a range of multiplexing approaches, discussed in depth previously^24^. Briefly, the models used here are individual-based and aim to capture a cage-trial type experiment; i.e. relatively low numbers of individuals, non-overlapping generations and no outside ecological factors such as density dependence. Within these models, stochastic effects are captured through either random selection (i.e. choosing individuals to seed the next generation and allocation of offspring genotypes) or comparing values randomly chosen from a distribution to selected parameter values (i.e. fitness costs, target site cleavage and repair via homing or NHEJ).

We also consider a number of modelling assumptions that are vital in moving from the values derived here to models of full classical or additive multiplexing approaches. Prior experience shows that the majority of gene drive elements impart a fitness cost on carrying individuals, even when inserted into putatively neutral sites. Thus, here we assume each gene drive element has a moderate fitness cost (*ε*) of 5% relative to wild type, applied multiplicatively such that heterozygote fitness is (1 − *ε*) and homozygote fitness (1 − *ε*)^2^. Over time the additive approach allows for the accumulation of multiple drive elements in a single allele. Thus as a base assumption we cap the maximum fitness cost imparted on an individual at (1 − *ε*)^2^ since the elements should be located close enough as to only disrupt a single genomic region. Our other main assumption centres on the rates of sgRNA target cleavage and drive inheritance for additive drive elements. Each of these elements would in reality have their own rates of target cleavage and inheritance, however for simplicity we assume here that each element (four elements to match the number in the classical approach) has the same drive parameters—considering two different scenarios based on the elements analysed above (*cd*^*g225*^ and *cd*^*g384*^). Full parameter sets are detailed in Materials and Methods.

**Fig. 6:**
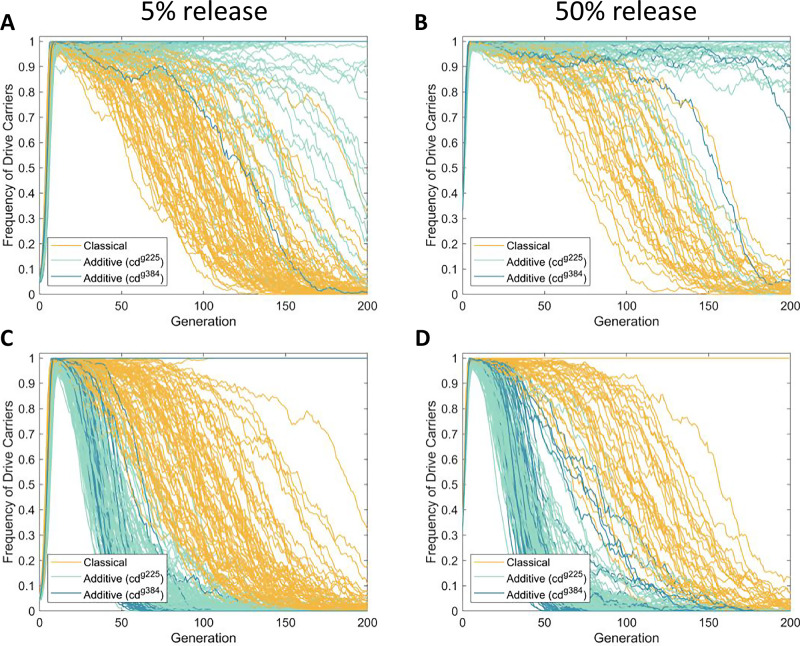
Additive multiplexing can outperform classical multiplexing so long as fitness costs are limited. Sample numerical simulations of the classical multiplexing (yellow) and two parameterisations of the additive multiplexing approach (*cd*^*g225*^ (green) and *cd*^*g384*^ (blue)). The top row (**A** and **B**) shows simulations for the scenario in which multiplicative fitness costs for the additive strategy are capped at the value for two copies while the bottom row (**C** and **D**) show simulations in which these multiplicative fitness costs are uncapped. The left column (**A** and **C**) displays simulations that consider an initial release of 0.05 times the initial wild population, whereas the right column (**B** and **D**) shows simulations for an initial release of 0.5 times the initial wild population. Note that each line represents a single stochastic numerical simulation and that many lines overlap one another at a drive carrier frequency of one as these represent all of the simulations in which resistance did not take hold.

Due to the stochastic nature of the models used here, we run 100 numerical simulations of the classical multiplexing model and the two additive model parameterisations (for *cd*^*g225*^ and *cd*^*g384*^) for each scenario considered (Fig. 6). Importantly, both multiplexing approaches are capable of attaining a high carrier frequency, with all simulations reaching a carrier frequency above 0.9 (i.e. >90% of individuals carry at least one drive copy) within ~10 generations. This holds for both parameterisations of the additive multiplex strategy (*cd*^*g225*^ and *cd*^*g384*^).

We first consider the base case in which the multiplicative fitness costs of the additive approach are capped at the value for two drive copies (Fig. 6A, B). Here we see that despite the classical and additive approaches reaching high frequency within a similar number of generations, both parameterisations of the additive approach (*cd*^*g225*^ and *cd*^*g384*^) outperform classical multiplexing in terms of their ability to persist within the population. Specifically, for the additive strategy based on *cd*^*g225*^ parameters a total of 10 out of 100 simulations had consistently fallen below a carrier frequency of 0.9 within the 200 generation window of simulation in the large release case, while 19 out of 100 had fallen below this threshold in the small release case. For the additive approach based on *cd*^*g384*^ parameters we saw only 2 and 1 simulations fall below this threshold for the large and small release cases, respectively. This is in stark contrast to the classical multiplexing approach, which showed 34 and 76 simulations falling below this threshold for the large and small release cases, respectively, with the vast majority of those dropping to near extinction within this time frame. The likely cause of this, as discussed previously^24^ is that, when all (or at least the outermost) sgRNAs cut simultaneously, the system has the potential to repair the region via NHEJ, resulting in the deletion of all target sites within a single generation. Within our previous study^24^, this was found to be the one of the most common causes of drive failure within the classical multiplexing approach. The additive approach, however, does not allow for such an event to occur, likely meaning that accumulation of resistance alleles is far slower, resulting in the improved performance (Fig. 6A, B).

Thus far we have considered a base scenario in which the fitness costs of the additive multiplexing approach is capped at that for two drive copies. This appears to be a reasonable assumption since each additive drive element should be located close enough together that they only disrupt one genomic region. However, another possibility is that by increasing the amount of disruption to that target region, or transgene expression, through the insertion of multiple additive drive elements may cause fitness costs to increase. Thus, we now progress to consider a scenario in which each additive drive element contributes to the multiplicative fitness cost (i.e. we remove the two element cap on fitness costs). Results of this represent a worst-case scenario in terms of the fitness costs imposed by an additive multiplex approach (Fig. 6C, D). These results clearly show that the additive approach can be substantially limited if multiple additive drive elements contribute equally to the overall fitness cost. For instance, removing the two element cap on fitness costs causes the number of simulations falling consistently below a drive carrier frequency of 0.9 within 200 generations to rise from 2 to 35 and 1 to 33 for the *cd*^*g384*^ parameterisation in the large and small release scenarios, respectively. Similarly, for the *cd*^*g225*^ parameterisation, the number of simulations falling below this threshold rises from 10 to 100 (i.e. all simulations) and 19 to 100 (i.e. all simulations) for the large and small release cases, respectively. Perhaps more importantly, removing the cap on fitness costs for the additive approach causes it to perform significantly worse than the classical multiplexing approach.

Put together, these results indicate that both the classical and additive multiplexing approaches have the potential to achieve rapid increases in frequency and a good degree of persistence within the population. While the additive approach can overcome a major issue of the classical approach (deletion of all target sites in a single generation), if each additive drive element contributes significantly to the overall fitness cost of carrier individuals, then the performance of this approach drops off drastically. Thus, these approaches show significant potential for further development, though further work is needed to fully understand the fitness characteristics of the additive approach.

## Discussion

Both population-suppression and population-modification gene drives are sensitive to resistance alleles. These alleles can be resistant to the drive and functional (r1) or non-functional (r2) alleles of the gene targeted. The first generation of population suppression homing gene drives generated in *An. gambiae* suffered from selection of a target site which was not functionally constrained^38^. It became clear that this target site coupled with high levels of Cas9 deposition created conditions which generated NHEJs and resulted in selection in favour of functional (r1) but drive resistant alleles in a caged population^17^. Population suppression drives aim to incur a deliberate fitness cost, as a result r1 resistance alleles will be positively selected for in a population, even if not fully functional, so long as they retain even a modest fitness advantage over the drive allele. As population modification drives are ideally neutral, either by insertion into a neutral locus or by restoring gene function if in a selected locus, resistance alleles (r1/r2) should not have a fitness advantage over the drive allele—in practice the drive allele likely has a modest cost, so the potential fitness advantage of resistance may be non-zero, but still lower than for a suppression drive. However, any kind of cut-resistant allele can result in drive failure for both population suppression and population modification gene drives if not mitigated. Strategies that aim to overcome resistance are critical for the success of homing-based gene drives as vector control strategies. Multiplexing has emerged as a promising way to mitigate target-site resistance by targeting multiple loci simultaneously^24,25,39–42^. In this study, we compare two multiplexing strategies to a single sgRNA drive and determine their homing and cleavage efficiencies in *An. stephensi*, investigating these strategies in depth in this mosquito species.

Using an endogenous *zpg-*expressed Cas9, the engineered classical multiplexing line (*cd*^*g338-384*^) showed over 99% germline cutting and inheritance bias, which was slightly, but not significantly, higher than the cutting and homing rates obtained with the singleplex drive (*cd*^*g384*^). Even though the *cd*^*g384_del*^ transgene utilises the most active sgRNA, this line showed the lowest cleavage rate and inheritance bias associated with an increased variance, potentially due to the requirement for resection for all homing events in this line (can be seen in the confidence intervals in Fig. 1B). These results suggest the addition of multiple sgRNAs in the classical multiplexing line are responsible for the increase in inheritance despite the required resection of the homology arms, which appears to increase the variability of cutting within individuals in the absence of additional sgRNAs. One of the potential challenges of the classical multiplexing strategy is when simultaneous double-stranded breaks at multiple sites are repaired via NHEJ, resulting in the deletion of the intervening region and creating resistance to all the target sites found within such deletion and potentially to the drive. Analysis of indel formation in trans-heterozygotes revealed that 14% of mutated sequences had lost all four sgRNA target sites under our classical multiplexing design. These rates are higher than previously reported in *Ae. aegypti*^43^, where no complete sgRNA deletions were observed in individuals carrying multiplexed sgRNA expression constructs, so our results represent a worst-case scenario, providing an upper-bound estimate of potential indel formation in the progeny. It should be noted that the present study sequenced indels as a result of somatic cutting and not germline. These data all highlight that resistance remains a critical consideration, and alternatives to the classical multiplexed gene drive systems are needed.

To overcome this limitation, alternative multiplexing strategies, including the additive multiplexing strategy, have been proposed^24^, but to date none have been assessed experimentally. Here, we assessed the efficiency of the additive multiplexing strategy to overcome resistance. Our results highlight the effectiveness of an additive multiplexing strategy, in which each singleplex drive element retains high inheritance rates, regardless of the presence of resistance alleles at the alternate target site(s). This redundancy offers a significant advantage over classical multiplexing strategies that could enable simultaneous cleavage at multiple sites within a single construct, where loss of one or more target sites can compromise drive efficiency. By distributing drive activity across separate loci, the additive design reduces the selective pressure for any one resistant allele to block transmission entirely. Importantly, the consistent performance of each singleplex element both in the presence and absence of resistance suggests that this strategy can provide enhanced stability and long-term efficacy, allowing for iterative releases to overcome any resistance alleles in a given population. Modelling in our previous work revealed that a potential downside of the additive strategy is that when multiple elements are present in a population homing could result in them becoming linked^24^. If both target sites are cut simultaneously an NHEJ event could result in a deletion of the intervening sequence. While this would likely result in a non-functional allele, in a neutral locus, such as might be used for population replacement drives, this new allele would be resistant to both elements. Another alternative strategy proposed is the “blocking” strategy^24^, similar in design to the *cd*^*g384_del*^ transgenics generated as a control for this study. In this strategy each construct expresses a single sgRNA and removes (blocks) adjacent target sites. Use of this strategy would eliminate the potential downfall of the classic strategy where a simultaneous cut at the outer two sgRNA targets yields an allele which is resistant to all sgRNAs in a single step. The potential downside to this strategy is the deletion of homologous sequences adjacent to the target site such that all cuts require resection for homing to occur. In *D. melanogaster* this has been shown to decrease drive efficiency, in some cases completely^29^. However, the high homing rates found with the *cd*^*g384_del*^ line, with only a 3% decrease from a construct expressing the same sgRNA but with perfect homology arms, indicate this strategy is feasible in *An. stephensi*. Note that the high homing rates in Anophelines may mask subtle effects of such sequence heterology observed in other mosquitoes^44,45^.

Mathematical modelling shows that both classical and additive multiplexing strategies demonstrate considerable promise for achieving high drive frequencies and persist within target populations, with the additive design presenting a slight advantage when fitness costs are capped at two alleles. However, our modeling also underscores a key limitation of the additive multiplexing strategy: its performance is highly sensitive to the cumulative fitness costs imposed by each individual drive element. If each allele contributes substantially to fitness reduction, the combined burden can significantly impede the spread and persistence of the drive in the target population and simultaneous targeting of multiple genes might not be compatible with the additive multiplexing strategy. This trade-off reflects a broader challenge in gene drive development, where the balance between drive efficacy and host fitness must be carefully optimised^16,43,46,47^. In contrast, classical multiplexing systems may impose lower cumulative costs by packaging multiple gRNAs within a single construct, though they remain vulnerable to large resistance-inducing deletions. Therefore, the choice between multiplexing strategies may depend on the specific biological and ecological context, including target species, population structure, and regulatory constraints. Importantly, future work should focus on appropriate target genes for the desired type of drive, experimentally quantifying the fitness costs associated with multi-element additive drives, particularly in heterozygous individuals, as well as their stability over multiple generations. Overall, both multiplexing strategies offer promising avenues for enhancing the robustness of homing-based gene drives, and further refinement of these approaches will be crucial to developing safe, effective, and confinable tools for the control of *An. stephensi* and other mosquito vectors of disease.

## Methods

### Maintenance of mosquito colony

*An. stephensi* of the SDA-500 wild-type strain and transgenics were reared under standard conditions of 70-80% relative humidity, 28 ± 1 ^o^C, and 14:10 h day-night cycle^48,49^. First instar larvae were fed with Sera Micron (Olibetta) and with Extra Select Pond Pellets Complete Fish Food at later stages. Adults were fed with 10% sucrose ad libitum and females were blood-fed with defibrinated horse blood (TCS Biosciences) administered through a Hemotek membrane feeding system (Hemotek, Inc) covered with a double layer of Parafilm (Bemis).

### Plasmid design and synthesis

AGG2072 (*cd*^*g384_del*^) was synthesised and contains ~1.5 kb homology arms, the Hr5/IE1-ZsGreen fluorescent marker and the *As*7SK regulatory regions^36^ expressing the sgRNA (GCAGCAACTTAATCAAGCCA).

AGG2301 (*cd*^*g338-384*^) was synthesised starting with the AGG2072 plasmid and adding three additional sgRNA expression cassettes utilising three previously characterised pol III promoters^36^ to express the additional sgRNAs (U6A-GGAGCGTACAAAGTTCATGC, U6B-TACGGATCACCTGGACCCAG and U6C-GGGAAACATTCCGGGTGTTG). It should be noted that due to their proximity in the genome, the region used in the reference for the U6A terminator overlaps with the U6B promoter region, this region has not been duplicated in the construct to avoid the potential for recombination. To avoid repetition of the sgRNA backbone, alternatives were selected based on a pilot experiment in Aag2 cells (Supplementary Fig. 4).

AGG2273 (*cd*^*g225*^) was synthesized and consists of ~1 kb homology arms, *An. gambiae cd* (AGAP003502) was used to replace *cd* coding sequence followed by a recoded ex6 of *An. stephensi cd*, and the AGAP003505 3’UTR. A cassette to express the sgRNA (CGCGCGTTAAAATCACACTG) consists of the *An stephensi* U6C regulatory regions^36^. An additional cassette contains the Hr5/IE1 promoter expressing the ZsGreen fluorescent marker protein. Due to poor fitness of this line, a second version of this construct was also made without the recoding (AGG2360).

Complete plasmid sequences are available through NCBI: AGG2072_cdg384_del: PV342346, AGG2360_cdg225:PV342348, AGG2301_cdg338-384:PV342349, AGG2273_cdg225: PV342347. Plasmids are available on request.

### Microinjection and screening of transgenic mosquitoes

Transgenic mosquitoes were generated by microinjection of embryos following a previously described protocol^48,49^. Briefly, adult mosquitoes reared under standard conditions but under a reversed light cycle, were blood fed with defibrinated horse blood (TCS Biosciences). 3–5 days after blood feeding, females were allowed to lay eggs in an egg collection cup for 45 min. The collected embryos were aligned, transferred into a coverslip using a double-sided tape (3 M), and covered with halocarbon oil 27 (Sigma). Embryo injections were performed using quartz capillaries (Sutter QF1007010) pulled with a P2000 laser pipette puller (Sutter) and a FemtoJet 4x microinjector (Eppendorf). Embryos were injected with 1× injection buffer^50^, 300 ng/μl of the donor plasmid (except for AGG2072 800 ng/μl was used), and 300 ng/μl of AGG1760 plasmid (NCBI:PQ306470) expressing Cas9 or Cas9 protein (PNABio). The injected embryos hatched approximately 24 h after injections and the G_0_ larvae were transferred to rearing trays where they were standard reared. G_0_ adults were pooled according to sex in groups of 20 with 20 G_0_ females crossed to 40 WT males or 20 G_0_ males crossed to 100 SDA-500 females. The offspring (G_1_) were screened for the presence of the fluorescent marker as larvae using a Leica MZ165FC microscope (Leica Biosystems). Positive G_1_ adults were individually outcrossed to SDA-500 to generate isolines. Once each isoline was secured, the G_1_ mosquitoes were collected for gDNA extraction, which was used as a template for PCR and sequence confirmation of the insertion site (Supplementary Fig. 5).

### Generation of *cd*^*225R*^ and *cd*^*384R*^ knock-out lines

Two different *cd* knock-out lines were generated to determine the cutting rate induced by Cas9. The *cd*^*225R*^ knock-out line was generated by crossing *zpg*^3’Cas9^ expressing females to *cd*^*g225*^ heterozygous males^36^. All the non-fluorescent offspring were intercrossed and blood fed. Their offspring were screened for a pink-eyed phenotype. The mutation of a single pink-eyed female was sequenced, showing a 1 bp deletion which results in a frame shift and a stop codon after amino acid 251. The generation of the *cd*^*384R*^ knock-out line was previously described^36^, where it was referred to as *cd*^*−/−*^.

### Generation of Cas9 lines with *cd*^*225R*^and *cd*^*384R*^ background

To determine the homing and cutting rates of the different sgRNA-expressing constructs in the presence of pre-existing target site resistance, fifty *zpg*^3’Cas9^ heterozygous males were crossed with fifty homozygous females of the *cd*^*384R*^ and *cd*^*225R*^ lines, to generate double heterozygous *zpg*^3’Cas9^;*cd*^*384R*^ and zpg^3’Cas9^*;cd*^*225R*^ lines. Subsequently, fifty double heterozygotes were then crossed to homozygous *cd*^*384*R^ or *cd*^*225R*^ lines. From those progeny, fifty pink-eyed males containing the *zpg*^3’Cas9^ transgene were selected and crossed to homozygous *cd*^*384R*^ or *cd*^*225R*^ females, and the line was maintained by repeating this cross every generation.

### Assessing the CRISPR/Cas9-induced homing and cutting rates

Fifty *zpg*^*3’Cas9*^ heterozygous females were crossed to fifty heterozygous males of the pertinent *cd* sgRNA expressing line (F_0_) to obtain trans-heterozygotes. Larvae with both transgene markers were sorted using a Biosorter (Union Biometrica). In addition, 200 non-sorted larvae were counted using the Biosorter. Sorted larvae were maintained under standard conditions and reared to adulthood. Non-sorted larvae were screened as L4 to obtain the ratios of the genotypes generated in the F_1_. Adult male trans-heterozygotes (F_1_) for the transgene expressing the sgRNA and the *zpg*^*3’Cas9*^ (or *zpg*^3’Cas9^;*cd*^*384R*^ or zpg^3’Cas9^*;cd*^*225R*^) allele were crossed in a 1:1 ratio to *cd* mutant females. The females were allowed to mate for a minimum of 5 days, when they were offered a blood meal. After 24 h, females were transferred into disposable espresso coffee cups (Somoplast) containing 30 mL of RO water, where they laid eggs. F_2_ larvae hatched and were maintained in the coffee cups until they were screened for fluorescence and eye phenotype under a Leica MZ165FC fluorescent stereo microscope (Leica Biosystems) as late larvae. The proportion of F_2_ larvae which inherited the transgene expressing the sgRNA(s) was used to determine the inheritance rates and the proportion of F_2_ larvae which presented a pink eyed phenotype was used to determine the cutting rate for *cd*^*g384_del*^ and *cd*^*g338-384*^. For line (*cd*^*g225*^) due to the recoding, heterozygotes will maintain a WT eye phenotype, so for crossing involving this line, the cleavage rate was calculated by adding the rate of inheritance of *cd*^*g225*^ to the number of mosaics in the non-*cd*^*g225*^ inheriting progeny.

### Amplicon sequencing and analysis

Genomic DNA of 10 F_1_ trans-heterozygous males of *zpg*^*3’Cas9*^*;cd*^*g338-384*^*, zpg*^*3’Cas9*^;*cd*^*g384*^, or *zpg*^*3’Cas9*^;*cd*^*g225*^ (AGG2360) and 10 SDA-500 males was extracted using the NucleoSpin Tissue kit (MachereyNagel). A 280 bp or 471 bp fragment surrounding the sgRNA target sites of the respective transgenic lines was amplified using primers listed in Supplementary Table 5. The amplicons were visualized by gel electrophoresis then purified using the NucleoSpin Gel and PCR Clean-up Kit (MachereyNagel) and sent for amplicon sequencing using the Illumina-based AMPLICON-EZ sequencing service from Genewiz (Leipzig, Germany). The sequencing reads obtained were analyzed using CRISPResso2 (Supplementary Table 6). The indel rates surrounding the sgRNA recognition sites were plotted using ggplot2 in R version 4.4.2.

#### Frameshift mutation analysis

In-frame and out-of-frame indels in modified sequences were determined by calculating if the number of nucleotides inserted or deleted is divisible by three using a formula in Microsoft Excel (formula: “=MOD((number of deletions - number of insertions), 3) = 0”). The proportion of frameshift indels in all reads with indels was calculated.

#### Resistance allele formation analysis

The rates of resistance allele formation in *cd*^*g338-384*^ were determined by counting the number of read sequences lacking the sequences of sgRNAs (1, 2, 3, and 4), as well as 3 m and 4 m (single SNP commonly found in wildtype in sgRNA 3 and 4), using the COUNTIF function in Microsoft Excel. The syntax and datasets used for analysis are available in the Source Data.

### Statistics and reproducibility

Analyses of inheritance ratios were carried out using R version 4.3.3^51^. A power analysis was used to predetermine approximate sample size prior to experiments also using R. Using the parameters 2 groups and an Alpha of 0.01, with an estimated standard deviation of 0.3, the progeny of at least 20 individuals would be necessary to determine a 20% difference in inheritance with a power of 0.8. With this in mind crosses were generated to assess at least this number. Estimated means and 95% confidence intervals were calculated by a Wald z-distribution approximation from a Generalized Linear Mixed Model (GLMM), with a binomial (“logit” link) error distribution fitted using the glmmTMB package^52^. Where applicable, initial parameters included the sgRNA transgenic line and the genetic background of the *zpg*^*3’Cas9*^*;cd*^*+/-*^ line; individual replicates were included as a random effect. The model with the best fit was then chosen by comparing Akaike Information Criterion (AIC) and summarized with “emmeans”^53^, model residuals were checked for violations of assumptions using the “DHARMa” package^54^. No data were excluded from the analyses. The experiments were not randomised. The Investigators were not blinded to allocation during the experiment and outcome assessment. Scripts and raw data can be found at (10.5281/zenodo.18877590).

### Modelling

Mathematical approaches used here are discussed extensively in our previous work^24^ with details and assumptions specific to this work detailed above. Simulations were conducted using MATLAB (version R2025a; The MathWorks Inc., Natick, MA) while plots were created using a combination of MATLAB versions R2025a and R2023a. Model scripts and associated output data are available via the Open Science Framework (https://osf.io/tsquv/).

Parameters used within the model scripts are as follows.

All approaches: Num_Targets = 4, Rel_Ratio = 0.50 or 0.05 (high and low release sizes), Num_Mosquitoes = 500, Num_Eggs = 15, Num_Generations = 200.

Classical: Prob_Cutting = 1, Prob_Home = 0.991, Prob_NHEJ = 1-Prob_Home, RelFit_Het = 0.95, RelFit_Hom = 0.9025, RelFit_WT=1.

Additive (*cd*^*g225*^): Prob_Cutting = 0.958, Prob_Home = 0.8785, Prob_NHEJ = 1-Prob_Home, RelFit_Het = 0.95, RelFit_WT = 1.

Additive (*cd*^*g384*^): Prob_Cutting = 1, Prob_Home = 0.9918, Prob_NHEJ = 1-Prob_Home, RelFit_Het = 0.95, RelFit_WT = 1.

### Reporting summary

Further information on research design is available in the Nature Portfolio Reporting Summary linked to this article.

## Supplementary information

Supplementary Information

Description of Additional Supplementary Files

Supplementary Data 1

Supplementary Data 2

Reporting Summary

Transparent Peer Review file

## Source data

Source Data

## Data Availability

All data generated for this manuscript is available in the manuscript, the supplemental files, or have been deposited in a public database. The AmpliconSeq raw data generated in this study have been deposited in NCBI: Bioproject PRJNA1269442. The sequences for plasmids generated in this study have been deposited to NCBI under Accession numbers: AGG2072_cdg384_del: PV342346, AGG2360_cdg225: PV342348, AGG2301_cdg338-384: PV342349. Chromatograms of Sanger sequencing to confirm the transgene insertions are available at 10.15124/d40a2165-fb3d-4458-9267-b6524858e6a8. Source data are provided with this paper.

## Code availability

Scripts for Crispresso2 analysis, statistical analysis and raw data can be found at (10.5281/zenodo.18877590) and are available under the MIT license. Model scripts and associated output data are available via the Open Science Framework (https://osf.io/tsquv/) under the MIT licence.
