# Supplementary material for: Integrating multiplexing into confineable gene drives effectively overrides resistance in Anopheles stephensi
Source: Nat Commun. 2026 May 7;17:6844. doi: 10.1038/s41467-026-72835-5 (PMC13388953; doi:10.1038/s41467-026-72835-5)
Supplement: Supplementary file 1 — Supplementary Information [file 41467_2026_72835_MOESM1_ESM.pdf]

Supplementary information

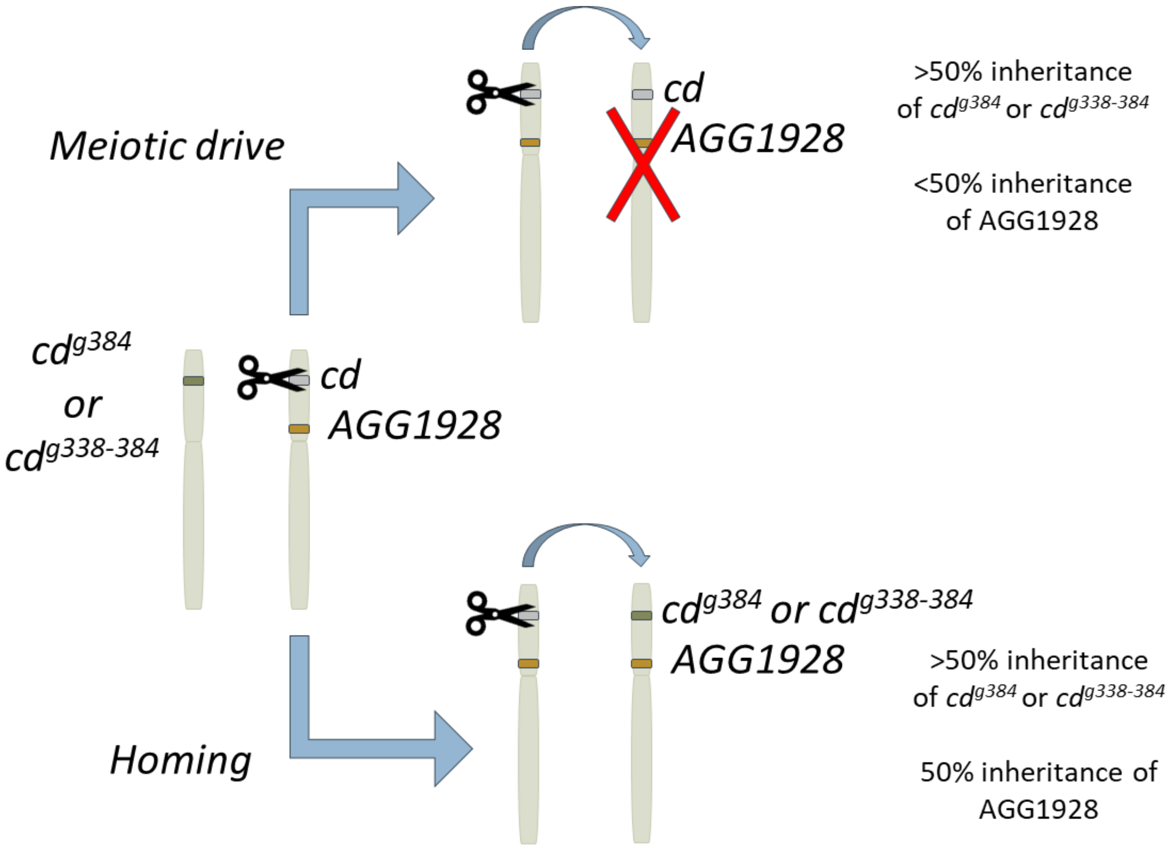

Supplementary Figure 1. Homing and meiotic drive can result in a biased inheritance. Schematic of the *cd* containing chromosome, with the cd^{g384} or $cd^{g338\text{-}384}$ transgene (green box) and the other chromosome containing the wt *cd* allele (grey box) and the AGG1928 transgene (yellow box). The recombination rate between AGG1928 and *cd* is <1%. CRISPR/Cas9 cleaves the wt *cd* allele (scissors) and if homing occurs the transgene is copied onto the AGG1928 marked chromosome, linking these two alleles. Alternatively, if meiotic drive occurs, the cut chromosome containing the wt *cd* allele and the AGG1928 transgene is removed from the gamete pool, which would result in less than Mendelian inheritance of the AGG1928 transgene, and a greater than Mendelian inheritance of the cd^{g384} or $cd^{g338\text{-}384}$ transgene.

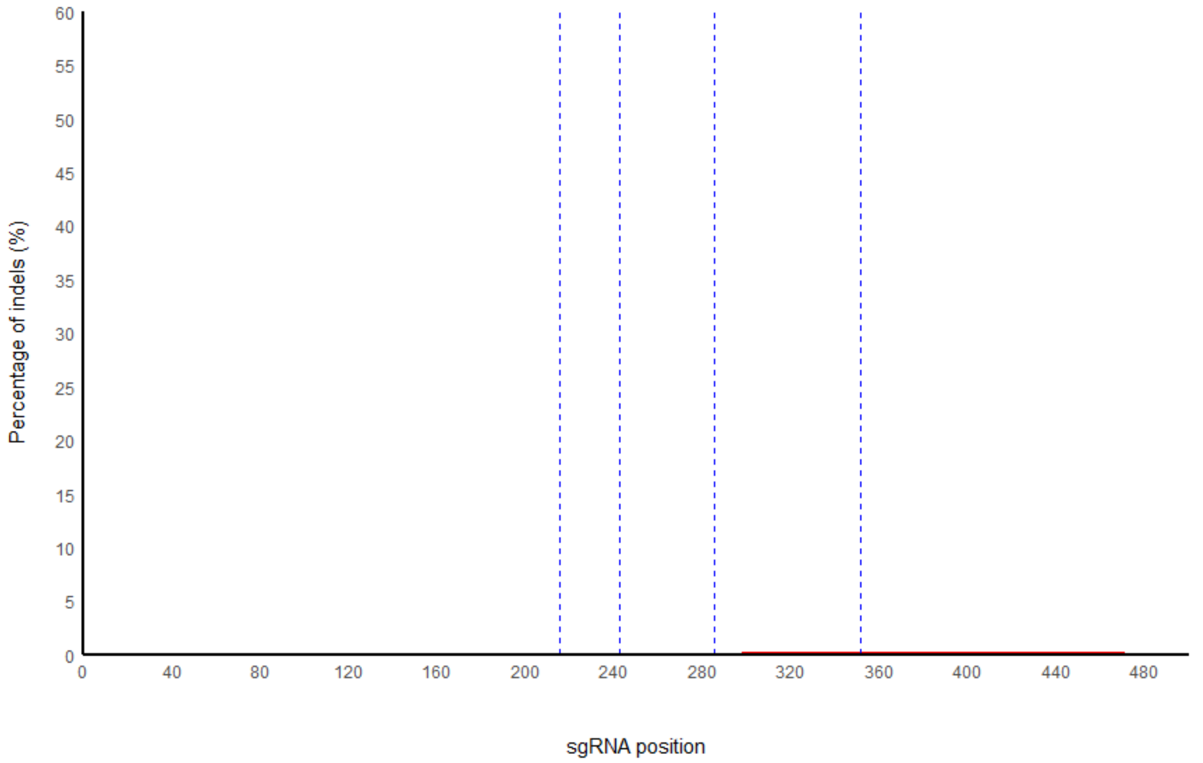

Supplementary Figure 2. Rates of insertions and deletions (indels) in the *cd* gene of WT males. Figure was obtained through CRISPResso2 and data can be found in the Source Data file.

```
GAGTGCTGTGATCCGGGGCAGCCCCAACACCCGGAATGTTTCCCAGTTCCGCTGGGTCCAGGTGATCCGTACTATCATCAGTACAATGTAACCTGCATGAACTTTGTACGCTCCGTACCGGCACCGACGGGTCATTTTGGTCCGCGGCAGCAACTTAATCAAGCCACGGCGTACATTGACGGCTCGGTTGT Reference
                            sgRNA338                     sgRNA347                                    sgRNA362                                                  sgRNA384

GAGTGCTGTGATCCGGGGC-------------------------------------------------------------------------------------------------------------------------------------------------------GTACATTGACGGCTCGGTTGT 0.45% (271 reads)
GAGTGCTGTGATCCGGGGCAGCCCCAACACCCGGAATGTTTCCCCGTTCCGCTGGGTCCGGGTGATCCGTACTATCATCAGTACAATGTAACCTGCATGAACTTTGTACGCTCCGTACCGGCACCGACGGGTCATTTTGGTCCGCGGCAGCAAC----------CACGGCGTACATTGACGGCTCGGTTGT 0.40% (244 reads)
GAGTGCTGTGATCCGGGGCAGCCCCAA----------------------------------------------------------------------------------------------------------------------------------------CCACGGCGTACATTGACGGCTCGGTTGT 0.34% (203 reads)
GAGTGCTGTGATCCGGGGCAGCCCCAACACCCGGAATGTTTCCCCGTTCCG---------------------------------------------------------------------------------------------------------------------GCGTACATTGACGGCTCGGTTGT 0.28% (170 reads)
GAGTGCTGTGATCCGGGGCAGCCCCAACACCCGGAATGTTTCCCCGTTCCGCTGGGTCCGGGTGATCCGTACTATCATCAGTACAATGTAACCTGCATGAACTTTGTACGCTCCGTACCGGCACCGACGGGTCATTTTGGTCCGCGGCAGCAACTTAA-----CCACGGCGTACATTGACGGCTCGGTTGT 0.26% (158 reads)
GAGTGCTGTGATCCGGGGCAGCCCCAACACCCGGAATGTTTCCCCGTTCCGCTGGGTCCGGGTGATCCGTACTATCATCAGTACAATGTAACCTGCATGAACTTTGTACGCTCCGTACCGGCACCGACGGGTCATTTTGGTCCGCGGCAGCAACTTAATC----CACGGCGTACATTGACGGCTCGGTTGT 0.25% (151 reads)
GAGTGCTGTGATCCGGGGCAGCCCCAACACCCGGAATGTTTCCCCGTTCCGCTGGGTCCGGGTGATCCGTACTATCATCAGTACAATGTAACCTGCATGAACTTTGTACGCTCCGTACCGGCACCGACGGGTCATTTTGGTCCGCGGCAGCAACTTAATCA-----CGGCGTACATTGACGGCTCGGTTGT 0.25% (149 reads)
GAGTGCTGTGATCCGGGGCAGCCCCAACACCCGGAATGTTTCCCCGTTCC-------------------------------------------------------------------------------------------------------------------ACGGCGTACATTGACGGCTCGGTTGT 0.25% (148 reads)
GAGTGCTGTGATCCGGGGCAGCCCCAACACCCGGAATGTTTCCCCGTTCCGCTGGGTCCGGGTGATCCGTACTATCATCAGTACAATGTAACCTGCATGAACTTTGTACGCTCCGTACCGGCACCGACGGGTCATTTTGGTCCGCGGCAGCAAC-------------GGCGTACATTGACGGCTCGGTTGT 0.25% (148 reads)
GAGTGCTGTGATCCGGGGCAGCCCCAACACCCGGAATGTTTCCCCGTTCCGCTGGGTCCGGGTGATCCGTACTATCATCAGTACAATGTAACCTGCATGAACTTTGTACGCTCCGTACCGGCACCGACGGGTCATTTTGGTCCGCGGCAGCAACTT-------CCACGGCGTACATTGACGGCTCGGTTGT 0.22% (135 reads)
```

Supplementary Figure 3. Ten most frequent mutations in ***cd***$^{g338\text{-}384}$**.** Alignment of the ten most common mutations, with sgRNA target sites underlined and PAM sites highlighted. Proportion of total reads is indicated as a percentage, with the number of reads that had that sequence indicated to the right of each sequence.

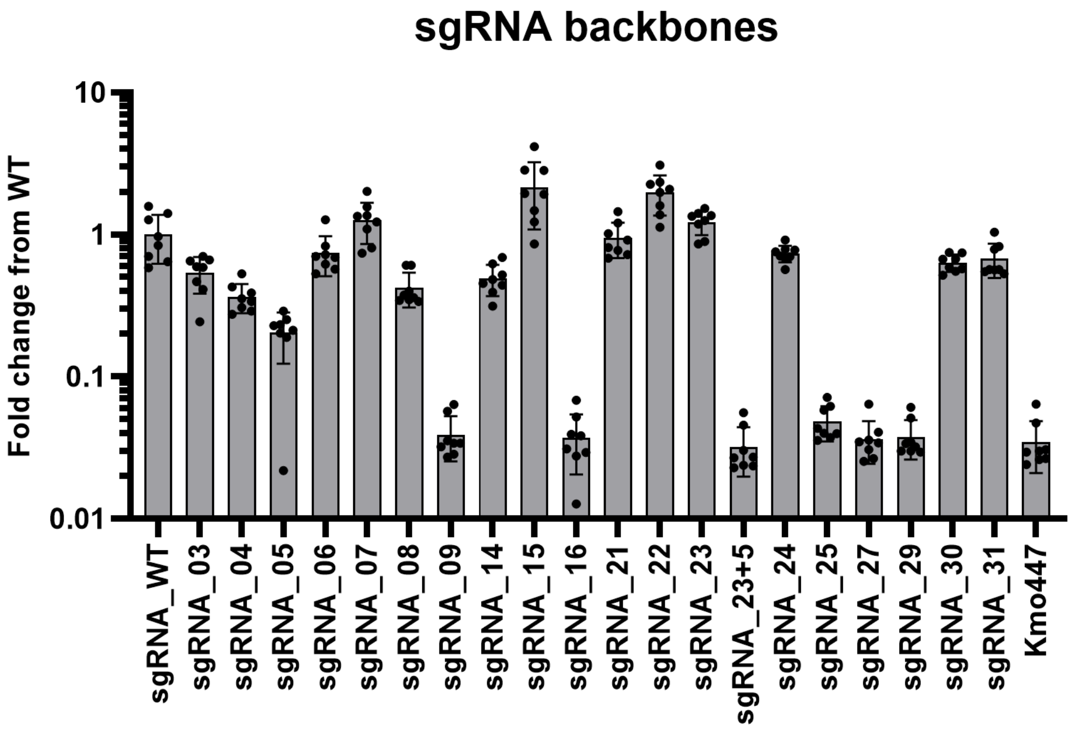

Supplementary Figure 4. dCas9-VPR assay [1] to determine suitability of different sgRNA backbone sequences [2] in mosquito cells. Aag2 cells were seeded in a 96 well plate and transfected using TransIT-Pro (Mirius) with 25ng dCas9-VPR, 25ng TRE-Firefly, 50ng pRL-OpIE2, 40ng *in vitro* transcribed sgRNA [3] per well, with 8 replicate wells per condition (n=8). After 48 hours cells were lysed in 1x Passive Lysis Buffer and the Dual luciferase assay performed on a GloMax Multi+ plate reader (Promega). Ratio of firefly to Renilla luciferase activity is normalised to the WT backbone sequence (sgRNA_WT). Kmo447 does not have a target site present in the reporter plasmid and is used to determine background.

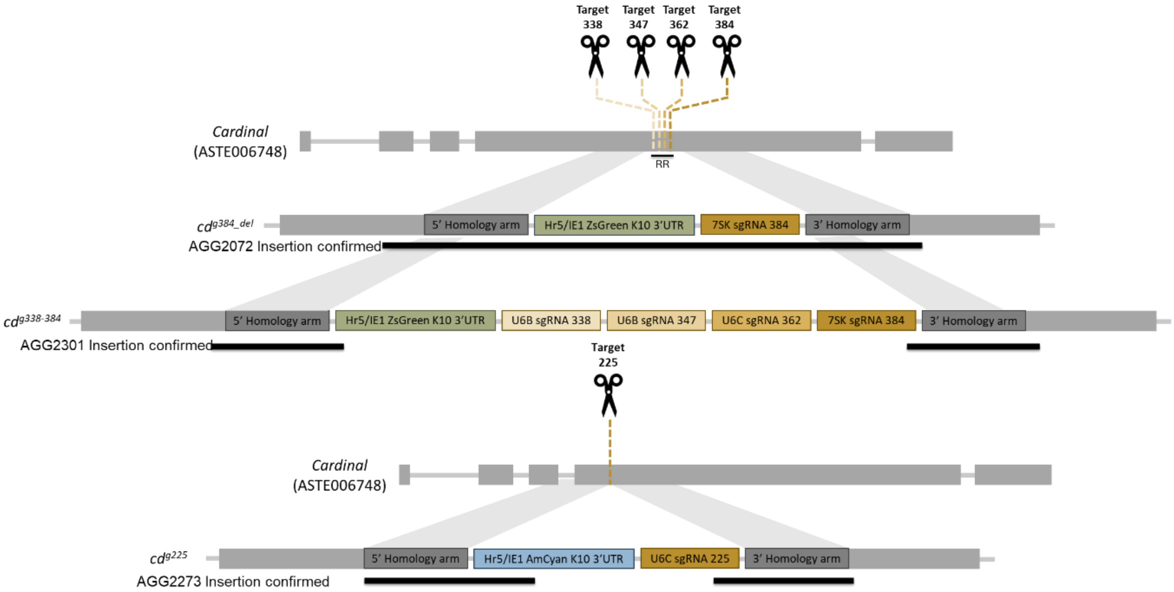

Supplementary Figure 5. PCR confirmation of the insertion site for all the generated lines. Black bars indicate regions which were amplified by PCR and Sanger sequence confirmed. Raw sequences are available at https://doi.org/10.15124/d40a2165-fb3d-4458-9267-b6524858e6a8.

Supplementary Table 1. Full model summaries for estimates of inheritance rates from sgRNAs. Log-odds and 95% confidence intervals, and significance values are taken from a binomial glmm with 'logit' link using sgRNA line and genetic background of the Cas9 bearing parent with full interaction terms (two-tailed). Mixed-effects models included replicate as a random factor.

	Inheritance Rate		
Predictors	***Odds Ratios***	***95% CI***	***P-Value***
(Intercept)	11.63	1.71 – 78.99	0.012
gRNA_typecd*g338-384*	18.95	1.22 – 293.69	0.035
gRNA type [cd*g384_del*]	2.36	0.22 – 24.75	0.475
gRNA type [cd*g384*]	15.02	1.71 – 132.34	0.015
pre cut [cd*225R*]	1.40	0.24 – 8.01	0.708
pre cut [cd*384R*]	0.82	0.05 – 12.49	0.886
gRNA_typecd*g338-384*:pre_cutcd*384R*	0.14	0.00 – 6.38	0.309
gRNA type [cd*g384*] × pre cut [cd*384R*]	0.01	0.00 – 0.24	0.006
**Random Effects**			
σ^2	4.93		
$\tau_{00\ id:group\_letter}$	1.64		
$\tau_{00\ group\_letter}$	0.86		
ICC	0.15		
N $_{id}$	328		
N $_{group\_letter}$	13		
Observations	328		
Marginal R^2 / Conditional R^2	0.325 / 0.425		

Supplementary Table 2. The inheritance bias observed for *cd*$^{g338\text{-}384}$ and *cd*^{g384} is due to homing and not meiotic drive. Full model summaries for estimates of inheritance rates from AGG1928 in crosses with *cd*^{g384} and *cd*$^{g338\text{-}384}$. Log-odds and 95% confidence intervals, and significance values are taken from a binomial glmm with 'logit' link error distribution using sgRNA line (two-tailed).

Predictors	Odds Ratios	95% CI	P-Value
**(Intercept)**	0.98	0.89 – 1.08	0.751
**gRNA type [cd$_{g384}$]**	0.95	0.84 – 1.08	0.434
**Observations**	32		

Transgenic	Estimate
***cd*$^{g338\text{-}384}$**	0.496 (0.472-0.52)
***cd*^{g384}**	0.83 (0.462-0.505)

Supplementary Table 3. Proportion of reads with out-of-frame and in-frame indels in in the F1 trans-heterozygous males of the *zpg*$^{3'Cas9}$;*cd*$^{g338\text{-}384}$, the *zpg*$^{3'Cas9}$;*cd*^{g384} , and the *zpg*$^{3'Cas9}$;*cd*^{g225}. Proportions are expressed in percentage of the reads with indels. Source data is provided as a Source Data file.

	Proportion of out-of-frame indels	Proportion of In-frame indels	Total number of reads with indels/total aligned reads
zpg$^{3'Cas9}$;*cd*$^{g338\text{-}384}$	70.06%	29.94%	33819/60438
zpg$^{3'Cas9}$;*cd*^{g384}	78.57%	21.43%	6630/10470
zpg$^{3'Cas9}$;*cd*^{g225}	71.72%	28.28%	9541/36536
SDA-500	72.29%	27.71%	83/66002

Supplementary Table 4. Proportion of reads lacking single to all sgRNAs of total modified reads and total reads in the F1 trans-heterozygous males of the *zpg*$^{3'Cas9}$;*cd*$^{g338\text{-}384}$, the *zpg*$^{3'Cas9}$;*cd*^{g384} , and the *zpg*$^{3'Cas9}$;*cd*^{g225}. Source data is provided as a Source Data file.

	Proportion of modified reads	Proportion of total reads
zpg$^{3'Cas9}$;*cd*$^{g338\text{-}384}$		
Lacking 1 sgRNA	44.2%	26.5%
Lacking 2 sgRNAs	25.6%	15.4%
Lacking 3 sgRNAs	15.6%	9.4%
Lacking 4 sgRNAs	14.2%	8.5%
Total	99.6%	59.8%
zpg$^{3'Cas9}$;*cd*^{g384}		
Lacking sgRNA384	100%	68.2%
zpg$^{3'Cas9}$;*cd*^{g225}		
Lacking sgRNA225	100%	29.1%

Supplementary Table 5. List of primers.

Primer name	Sequence	Purpose
LA247	CGATTGATGAGTCATTTGTT	PCR and Sequencing of cd^{g384_del}
LA299	GGTAACCGAGACAATGGAGAAGCAAGAG	PCR and Sequencing of cd^{g384_del}
LA4330	CGCAGCCGAAACGTGTTCAACATTC	PCR and Sequencing of $cd^{g338\text{-}384}$, cd^{g384_del}, cd^{g225}
LA4331	AATGCGTTGTATCAAATGACACGGCG	PCR and Sequencing of cd^{g384_del}, cd^{g225}
LA4332	GTCTGTTTAAACTTCACCGTCTTTCTGGG	PCR and Sequencing of cd^{g225}
LA4333	AGTTAAACCCGGACTATGGTGATGGC	PCR and Sequencing cd^{384R}, cd^{g384_del}, cd^{g225}
LA4335	CCGTGGCTTGATTAAGTTGCTGCC	Sequencing cd^{225R}
LA4336	GTACAGCGAGTAAGGATTGAACAGCATCC	PCR of cd^{384}, cd^{g225}
LA4337	CAGATAATGCTCGACAGCTTTGTGCC	PCR and Sequencing of cd^{g384_del}
LA4338	AGTACATACATTCTCAACCGAAGGCGC	PCR and Sequencing of $cd^{g338\text{-}384}$, cd^{g384_del}
LA4902	CGTTATCAACTTGAAAAAGTGGC	PCR and Sequencing of $cd^{g338\text{-}384}$, cd^{g384_del}
LA5160	GACCCAAGAAAAAGCGGAAGGTGG	PCR and Sequencing of cd^{g384_del}
LA5986	ATCAAGCTCCTCTAGATCCGGTGGATCTTAC	PCR and Sequencing of cd^{g384_del}
LA6101	CTGACCAAGGAGATGACCATGAAGTACCGCATG	PCR and Sequencing of cd^{g384_del}
LA6731	GGTAACCGAGACAATGGAGAAGCAAGAG	PCR and Sequencing of cd^{g225}
LA6791	CCAGTTCGGTTATGAGCCGT	PCR and Sequencing of cd^{g225}
LA6792	AATGACATCATCCACTGATCG	PCR and Sequencing of $cd^{g338\text{-}384}$
LA7592	CTCGGACGACGAGCGTATGG	PCR and Sequencing of cd^{g225}
LA7953	ATTGAAGGCCCGCGATAGACTC	PCR and Sequencing of cd^{g225}
LA8538	TAGCGGCCAAGGTGGGATTTGTGGAGGATCGTGCTAC	PCR and Amplicon Sequencing of $zpg^{3'Cas9}$;cd^{g225}
LA9133	TCGACGGAAGCACGTGGCGCCGAAA	PCR and Amplicon Sequencing of $zpg^{3'Cas9}$;cd^{g225}
LA8682	TTTCGGCGCCACGTGCTTCCGTCGA	PCR and Amplicon Sequencing of $zpg^{3'Cas9}$;$cd^{g338\text{-}384}$, $zpg^{3'Cas9}$;cd^{g384}, SDA-500
LA8684	GCAGCTGTCGACCGTCCGGTGTT	PCR and Amplicon Sequencing of $zpg^{3'Cas9}$;$cd^{g338\text{-}384}$, $zpg^{3'Cas9}$;cd^{g384}, SDA-500

Supplementary Table 6. Number of reads for each sample analysed by CRISPResso2.

Sample name	Reads in inputs	Reads after preprocessing	Reads aligned
zpg$^{3'Cas9}$;*cd*$^{g338\text{-}384}$	66158	63256	60438
SDA-500	71533	68186	66002
zpg$^{3'Cas9}$;*cd*^{g384}	45512	42529	10470
zpg$^{3'Cas9}$;*cd*^{g225}	47622	41612	36536

References

1. Anderson, M. A. E. *et al.* Expanding the CRISPR Toolbox in Culicine Mosquitoes: In Vitro Validation of Pol III Promoters. *ACS Synth. Biol.* **9**, 678–681 (2020).
2. Noble, C. *et al.* Daisy-chain gene drives for the alteration of local populations. *Proc. Natl. Acad. Sci.* **116**, 8275–8282 (2019).
3. Bassett, A. R., Tibbit, C., Ponting, C. P. & Liu, J.-L. Highly Efficient Targeted Mutagenesis of *Drosophila* with the CRISPR/Cas9 System. *Cell Rep.* **4**, 220–228 (2013).
