# Supplementary material for: Integrating multiplexing into confineable gene drives effectively overrides resistance in Anopheles stephensi
Source: Nat Commun. 2026 May 7;17:6844. doi: 10.1038/s41467-026-72835-5 (PMC13388953; doi:10.1038/s41467-026-72835-5)
Supplement: Supplementary file 2 — Description of Additional Supplementary Files [file 41467_2026_72835_MOESM2_ESM.pdf]

Description of Additional Supplementary Files

File Name: Supplementary Data 1
Description: Summary of crosses and expected genotypes, fluorescent markers and eye phenotypes

File Name: Supplementary Data 2
Description: Pairwise contrasts (with tukey adjusted P-values) for all sgRNA lines and genetic backgrounds derived from a fitted generalized binomial mixed-effects model (see Supplementary Table 1).
