# Supplementary material for: Integrating multiplexing into confineable gene drives effectively overrides resistance in Anopheles stephensi
Source: Nat Commun. 2026 May 7;17:6844. doi: 10.1038/s41467-026-72835-5 (PMC13388953; doi:10.1038/s41467-026-72835-5)
Supplement: Supplementary file 5 — Reporting Summary [file 41467_2026_72835_MOESM5_ESM.pdf]

Corresponding author(s): Michelle Anderson
Luke Alphey

Last updated by author(s): Mar 5, 2026

Reporting Summary

Nature Portfolio wishes to improve the reproducibility of the work that we publish. This form provides structure for consistency and transparency in reporting. For further information on Nature Portfolio policies, see our Editorial Policies and the Editorial Policy Checklist.

Statistics

For all statistical analyses, confirm that the following items are present in the figure legend, table legend, main text, or Methods section.

n/a	Confirmed	
☐	☒	The exact sample size (*n*) for each experimental group/condition, given as a discrete number and unit of measurement
☐	☒	A statement on whether measurements were taken from distinct samples or whether the same sample was measured repeatedly
☐	☒	The statistical test(s) used AND whether they are one- or two-sided<br>*Only common tests should be described solely by name; describe more complex techniques in the Methods section.*
☒	☐	A description of all covariates tested
☒	☐	A description of any assumptions or corrections, such as tests of normality and adjustment for multiple comparisons
☐	☒	A full description of the statistical parameters including central tendency (e.g. means) or other basic estimates (e.g. regression coefficient) AND variation (e.g. standard deviation) or associated estimates of uncertainty (e.g. confidence intervals)
☒	☐	For null hypothesis testing, the test statistic (e.g. *F*, *t*, *r*) with confidence intervals, effect sizes, degrees of freedom and *P* value noted<br>*Give P values as exact values whenever suitable.*
☒	☐	For Bayesian analysis, information on the choice of priors and Markov chain Monte Carlo settings
☒	☐	For hierarchical and complex designs, identification of the appropriate level for tests and full reporting of outcomes
☒	☐	Estimates of effect sizes (e.g. Cohen's *d*, Pearson's *r*), indicating how they were calculated

Our web collection on statistics for biologists contains articles on many of the points above.

Software and code

Policy information about availability of computer code

Data collection	no software used
Data analysis	We used R v. 4.3.3 (R Core Team 2024) and the following R packages: arm v. 1.13.1 (Gelman and Su 2022), brglm v. 0.7.2 (Kosmidis 2021; Kosmidis and Firth 2021), DHARMa v. 0.4.6 (Hartig 2022), emmeans v. 1.10.6 (Lenth 2024), glue v. 1.8.0 (Hester and Bryan 2024), gt v. 0.11.1 (Iannone et al. 2024), scales v. 1.4.0 (Wickham, Pedersen, and Seidel 2025), showtext v. 0.9.6 (Qiu and See file AUTHORS for details. 2023), sjPlot v. 2.8.16 (Lüdecke 2024), viridis v. 0.6.5 (Garnier et al. 2024), glmmTMB v. 1.1.8 (Brooks et al. 2017).<br>MATLAB version R2025a code available at https://osf.io/tsquv/<br>CRISPResso2 v. 2.0.20b

For manuscripts utilizing custom algorithms or software that are central to the research but not yet described in published literature, software must be made available to editors and reviewers. We strongly encourage code deposition in a community repository (e.g. GitHub). See the Nature Portfolio guidelines for submitting code & software for further information.

Data

Policy information about availability of data

All manuscripts must include a data availability statement. This statement should provide the following information, where applicable:

- Accession codes, unique identifiers, or web links for publicly available datasets
- A description of any restrictions on data availability
- For clinical datasets or third party data, please ensure that the statement adheres to our policy

All data generated for this manuscript is available in the manuscript, the supplemental files, or have been deposited in a public database. The AmpliconSeq raw data generated in this study have been deposited in NCBI: Bioproject PRJNA1269442. The sequences for plasmids generated in this study have been deposited to NCBI under Accession numbers: AGG2072_cdg384_del: PV342346 (https://www.ncbi.nlm.nih.gov/nuccore/PV342346.1/), AGG2360_cdg225:PV342348 (https://www.ncbi.nlm.nih.gov/nuccore/PV342348), AGG2301_cdg338-384:PV342349 (https://www.ncbi.nlm.nih.gov/nuccore/PV342349). Chromatograms of Sanger sequencing to confirm the transgene insertions are available at doi: https://doi.org/10.15124/d40a2165-fb3d-4458-9267-b6524858e6a8.

Research involving human participants, their data, or biological material

Policy information about studies with human participants or human data. See also policy information about sex, gender (identity/presentation), and sexual orientation and race, ethnicity and racism.

Reporting on sex and gender	n/a
Reporting on race, ethnicity, or other socially relevant groupings	n/a
Population characteristics	n/a
Recruitment	n/a
Ethics oversight	n/a

Note that full information on the approval of the study protocol must also be provided in the manuscript.

Field-specific reporting

Please select the one below that is the best fit for your research. If you are not sure, read the appropriate sections before making your selection.

☒ Life sciences ☐ Behavioural & social sciences ☐ Ecological, evolutionary & environmental sciences

For a reference copy of the document with all sections, see nature.com/documents/nr-reporting-summary-flat.pdf

Life sciences study design

All studies must disclose on these points even when the disclosure is negative.

Sample size	A basic power analysis was performed and determined that for 0.8 power, we should analyse progeny from at least 20 parents for a 20% difference between inheritance rates.
Data exclusions	No data were excluded.
Replication	Biological replicates were performed as described in the main article text. Insects with the same genotypes were crossed en mass and the progeny of each individual scored as biological replicates. The data for all replicates was included in analysis.
Randomization	The Biosorter was used to randomly sort 200 larvae of relevant genotypes into trays for rearing. These were then all used for crossing schemes as described in the text. All progeny of crossed individuals were screened for genotypes and phenotypes as stated in the text.
Blinding	Blinding in the traditional sense is not relevant to the study as there are no treated/untreated groups. All progeny from all females are scored and included in the analysis.

Reporting for specific materials, systems and methods

We require information from authors about some types of materials, experimental systems and methods used in many studies. Here, indicate whether each material, system or method listed is relevant to your study. If you are not sure if a list item applies to your research, read the appropriate section before selecting a response.

Materials & experimental systems

n/a	Involved in the study
☒	☐ Antibodies
☐	☒ Eukaryotic cell lines
☒	☐ Palaeontology and archaeology
☐	☒ Animals and other organisms
☒	☐ Clinical data
☒	☐ Dual use research of concern
☒	☐ Plants

Methods

n/a	Involved in the study
☒	☐ ChIP-seq
☒	☐ Flow cytometry
☒	☐ MRI-based neuroimaging

Eukaryotic cell lines

Policy information about cell lines and Sex and Gender in Research

Cell line source(s)	Aag2 cells were derived from newly hatched Aedes aegypti larvae. Sex is unknown as they do not appear to express genes involved in sex determination
Authentication	Aag2 cells have been verified to be Aedes aegypti by COI barcoding
Mycoplasma contamination	Cell lines tested negative for mycoplasma
Commonly misidentified lines (See ICLAC register)	n/a

Animals and other research organisms

Policy information about studies involving animals; ARRIVE guidelines recommended for reporting animal research, and Sex and Gender in Research

Laboratory animals	Anopheles stephensi SDA-500 strain obtained from Dr Andrew Blagborough at Cambridge University, males and females of all ages and life stages
Wild animals	n/a
Reporting on sex	Data for homing and cutting rate of males and females is reported separately.
Field-collected samples	n/a
Ethics oversight	No ethical oversight is required for insect species. All work is approved by the Biological Agents and Genetic Modification Safety Committee at The University of York.

Note that full information on the approval of the study protocol must also be provided in the manuscript.

Plants

Seed stocks	n/a
Novel plant genotypes	n/a
Authentication	n/a
