# Supplementary material for: Integrating multiplexing into confineable gene drives effectively overrides resistance in Anopheles stephensi
Source: Nat Commun. 2026 May 7;17:6844. doi: 10.1038/s41467-026-72835-5 (PMC13388953; doi:10.1038/s41467-026-72835-5)
Supplement: Supplementary file 6 — Transparent Peer Review file [file 41467_2026_72835_MOESM6_ESM.pdf]

Peer Review File

Integrating multiplexing into confineable gene drives effectively overrides resistance in *Anopheles stephensi*

Corresponding Author: Ms Michelle Anderson

Parts of this Peer Review File have been redacted as indicated to remove third-party material.

This file contains all reviewer reports in order by version, followed by all author rebuttals in order by version.

Version 0:

Reviewer comments:

Reviewer #1

(Remarks to the Author)
This study examines the use of different multiplex formulations (i.e. multiple gRNA cassettes) in Cas9-based homing gene drives in Anopheles stephensi. As seen previously in other Anopheles species, homing rates are high and substantially greater than those typically achieved in Aedes and other genera, where drive is more challenging. Multiplex formulations also mitigate the problem of target site mutations that would otherwise block single-gRNA drives. The experimental work is carefully executed and thoughtful. That said, the novelty and impact of the findings are somewhat limited, and the way the paper is currently written does not help make the case.

Where the clearest advance lies is in the experimental evaluation of different multiplex designs and the nuanced differences between them. This area—previously addressed in theoretical work by some of the same authors—benefits from the experimental exploration presented here, and reframing the paper to emphasise these aspects could strengthen the argument for novelty.

Some results sections are difficult to follow. Both I and a member of my laboratory found that the exposition demands considerable prior knowledge of experimental set-ups, cross designs, or previously described strains. This could be clarified to make the work more accessible. We have attached an annotated version of the manuscript with specific comments.

Beyond those detailed notes, two broader points are worth highlighting. First, the caveats around resistance allele formation vary depending on the type and intended purpose of the drive (e.g. suppression vs replacement). As written, the text risks confusing readers who are familiar with the concept of gene drive but not expert in the details. Greater care in distinguishing these contexts would improve clarity. Second, more transparency is needed on the starting assumptions in the additive multiplex approach. If drive elements are separate initially or become separated subsequently, what happens when they meet across homologues? Presumably they enter linkage, but the effects of potential cleavage across both chromosomes could be explored experimentally.

Reviewer #2

(Remarks to the Author)
I co-reviewed this manuscript with one of the reviewers who provided the listed reports. This is part of the Nature Communications initiative to facilitate training in peer review and to provide appropriate recognition for Early Career Researchers who co-review manuscripts.

Reviewer #3

(Remarks to the Author)
In their manuscript entitled “Integrating multiplexing into confineable gene drives effectively overrides resistance in Anopheles stephensi” Godall and colleagues describe a new gene drive approach in the pernicious malaria vector Anopheles stephensi.

The introduction is succinct and compelling, presenting a clear overview of gene drives, classical multiplexing strategies, and the newer variants e.g. ‘additive’ approaches that utilize multiple homing constructs. The experiments are thorough, the

results clearly presented (though see comments below), and the advance is important, providing guidance to the dozens of laboratories interested in generating effective population-modifying or -suppressing gene drives targeting disease-vectoring mosquitoes. The modelling work is clearly described and reinforces the intuition garnered through the experiments at the cd locus while adding additional considerations regarding 'additive' fitness costs that might comes with 'additive' gene drives.

I primarily have comments about data visualization outlined below that I feel would help make the manuscript easier to read and digest. Otherwise I think it is an important contribution to an important field and enjoyed reading it.

Major comments

1) Figure 5 is very difficult to parse - I would recommend an upSet plot (e.g. https://upset.app/) to help visualize the overlap between deletions at the different cut sites
2) Lines 414-415: The statement "It should be noted that this experiment sequenced indels as a result of somatic cutting and not germline, but we expect the same range of mutations to be generated" is intriguing to me - if there is data out there to back up this expectation it should be cited. In contrast, I can easily imagine differences in the ranges of mutations that are formed in the germline vs. somatic tissues depending on differential regulation or expression of repair machinery (although my hunch is also not backed up by data in insects). In any event, I would be happy to see any appropriate citations included here or the statement tempered to simply state the caveat that sequencing represents somatic rather than germ line mutagenesis

Minor comments

1) Figure 1B, 2B, 3B, I feel that the y axis should be more specific - 'individuals scored' is ambiguous as to the measured phenotype and should be more clearly described in the figure itself
2) I found the nomenclature of the cd^384 and cd^225 alleles to be confusing - perhaps cd^384Δ3 or cd^384R or something similar that intrinsically signals that it carries a small deletion or is resistant to sgRNA cleavage?
3) The x-axis in the panels of figure 4 are not labeled (or relevant) — I would recommend revising to make them relative to predicted cut site (e.g. zero at the dashed vertical line)

Version 1:

Reviewer comments:

Reviewer #1

(Remarks to the Author)
Due to a clerical mishap, it appears that the authors did not receive the annotated manuscript that accompanied my initial review. Together with an ECR in my group, I have re-evaluated the revised manuscript. While some issues were addressed, a number remain outstanding. We have therefore added the comments and suggested edits that are still relevant to the latest version provided. We hope these will be helpful.

The most substantive point in the original review concerned the framing around novelty. This remains largely unaddressed. In particular, the abstract is essentially unchanged, and the elements highlighted there, as written, do not convey a clear or novel contribution. Strengthening this framing would substantially improve the manuscript.To be clear, I am in favour of publication; my concern relates only to the clarity of the framing, not the suitability of the work

Reviewer #2

(Remarks to the Author)
I co-reviewed this manuscript with one of the reviewers who provided the listed reports. This is part of the Nature Communications initiative to facilitate training in peer review and to provide appropriate recognition for Early Career Researchers who co-review manuscripts.

Reviewer #3

(Remarks to the Author)
In their revised manuscript, Larossa-Godall and colleagues have addressed comments from reviewers ranging from data visualization issues (the updated figure 5 is much more straightforward to parse, in my opinion), to increasing the description of crossing schemes in the results and implications for field-based gene drive performance in the discussion. I have no further comments.

Version 2:

Reviewer comments:

Reviewer #1

(Remarks to the Author)
I am happy with the reviewers' replies to comments.

I believe Figure 5 should say "each sgRNA target site" not "each sgRNA"

Reviewer #2

(Remarks to the Author)
I co-reviewed this manuscript with one of the reviewers who provided the listed reports. This is part of the Nature Communications initiative to facilitate training in peer review and to provide appropriate recognition for Early Career Researchers who co-review manuscripts.

Open Access This Peer Review File is licensed under a Creative Commons Attribution 4.0 International License, which permits use, sharing, adaptation, distribution and reproduction in any medium or format, as long as you give appropriate credit to the original author(s) and the source, provide a link to the Creative Commons license, and indicate if changes were made.
In cases where reviewers are anonymous, credit should be given to 'Anonymous Referee' and the source.
The images or other third party material in this Peer Review File are included in the article's Creative Commons license, unless indicated otherwise in a credit line to the material. If material is not included in the article's Creative Commons license and your intended use is not permitted by statutory regulation or exceeds the permitted use, you will need to obtain permission directly from the copyright holder.
To view a copy of this license, visit https://creativecommons.org/licenses/by/4.0/

Response to Reviewers comments: NCOMMS-25-58127A "Integrating multiplexing into confinable gene drives effectively overrides resistance in *Anopheles stephensi*"

We would like to take the time to thank the editor and reviewers for their time and thoughtful comments which have helped to improve the manuscript and allows us to present our findings in a clear and thoughtful manner. Please find our point-by-point response to the reviewers comments in italics below.

REVIEWER COMMENTS

Reviewer #1 (Remarks to the Author):

This study examines the use of different multiplex formulations (i.e. multiple gRNA cassettes) in Cas9-based homing gene drives in Anopheles stephensi. As seen previously in other Anopheles species, homing rates are high and substantially greater than those typically achieved in Aedes and other genera, where drive is more challenging. Multiplex formulations also mitigate the problem of target site mutations that would otherwise block single-gRNA drives. The experimental work is carefully executed and thoughtful. That said, the novelty and impact of the findings are somewhat limited, and the way the paper is currently written does not help make the case.

Where the clearest advance lies is in the experimental evaluation of different multiplex designs and the nuanced differences between them. This area—previously addressed in theoretical work by some of the same authors—benefits from the experimental exploration presented here, and reframing the paper to emphasise these aspects could strengthen the argument for novelty.

Some text has been added/changed throughout the manuscript to address this comment.

Some results sections are difficult to follow. Both I and a member of my laboratory found that the exposition demands considerable prior knowledge of experimental set-ups, cross designs, or previously described strains. This could be clarified to make the work more accessible. We have attached an annotated version of the manuscript with specific comments.

Although the annotated version of the manuscript was not received, we have included additional explanation to further explain the crosses and previously described strains.

Beyond those detailed notes, two broader points are worth highlighting. First, the caveats around resistance allele formation vary depending on the type and intended purpose of the drive (e.g. suppression vs replacement). As written, the text risks confusing readers who are familiar with the concept of gene drive but not expert in the details. Greater care in distinguishing these contexts would improve clarity. Second, more transparency is needed on the starting assumptions in the additive multiplex approach. If drive elements are separate initially or become separated subsequently, what happens when they meet across homologues? Presumably they enter linkage, but the effects of potential cleavage across both chromosomes could be explored experimentally.

We have added a paragraph to the additive multiplexing results section to explain this system in more detail. The discussion has been expanded to include further explanation how resistance alleles can differentially affect population suppression and replacement drives as well as the potential for linkage in the additive strategy. While we agree with the reviewer that this could be explored experimentally, ultimately this strategy is likely not the ideal for field use and so we have not investigated this further. We also discuss the merits of the 'blocking' strategy in the discussion,

this alternative multiplexing strategy also explored theoretically in our previous works, prevents the issue of linkage and does seem feasible in An. stephensi, as is demonstrated by the high inheritance rates we observed in the cd^{g384_del} transgenics.

Reviewer #2 (Remarks to the Author):

I co-reviewed this manuscript with one of the reviewers who provided the listed reports. This is part of the Nature Communications initiative to facilitate training in peer review and to provide appropriate recognition for Early Career Researchers who co-review manuscripts.

Reviewer #3 (Remarks to the Author):

In their manuscript entitled "Integrating multiplexing into confineable gene drives effectively overrides resistance in Anopheles stephensi" Godall and colleagues describe a new gene drive approach in the pernicious malaria vector Anopheles stephensi.

The introduction is succinct and compelling, presenting a clear overview of gene drives, classical multiplexing strategies, and the newer variants e.g. 'additive' approaches that utilize multiple homing constructs. The experiments are thorough, the results clearly presented (though see comments below), and the advance is important, providing guidance to the dozens of laboratories interested in generating effective population-modifying or -suppressing gene drives targeting disease-vectoring mosquitoes. The modelling work is clearly described and reinforces the intuition garnered through the experiments at the cd locus while adding additional considerations regarding 'additive' fitness costs that might comes with 'additive' gene drives.

I primarily have comments about data visualization outlined below that I feel would help make the manuscript easier to read and digest. Otherwise I think it is an important contribution to an important field and enjoyed reading it.

Major comments

1) Figure 5 is very difficult to parse - I would recommend an upSet plot (e.g. https://upset.app/) to help visualize the overlap between deletions at the different cut sites

As suggested an upset plot has been generated to hopefully present the data in a clearer way.

2) Lines 414-415: The statement "It should be noted that this experiment sequenced indels as a result of somatic cutting and not germline, but we expect the same range of mutations to be generated" is intriguing to me - if there is data out there to back up this expectation it should be cited. In contrast, I can easily imagine differences in the ranges of mutations that are formed in the germline vs. somatic tissues depending on differential regulation or expression of repair machinery (although my hunch is also not backed up by data in insects). In any event, I would be happy to see any appropriate citations included here or the statement tempered to simply state the caveat that sequencing represents somatic rather than germ line mutagenesis

This statement has been tempered as this is an assumption on our part - as the reviewer suggests there is no data in insects to indicate that is or is not the case.

Minor comments

1) Figure 1B, 2B, 3B, I feel that the y axis should be more specific - 'individuals scored' is ambiguous as to the measured phenotype and should be more clearly described in the figure itself

The y-axis labels have been more clearly defined.

2) I found the nomenclature of the cd^384 and cd^225 alleles to be confusing - perhaps cd^384Δ3 or cd^384R or something similar that intrinsically signals that it carries a small deletion or is resistant to sgRNA cleavage?

The nomenclature has been changed, as suggested, to cd^{384R} *or* cd^{225R} *for clarification.*

3) The x-axis in the panels of figure 4 are not labeled (or relevant) — I would recommend revising to make them relative to predicted cut site (e.g. zero at the dashed vertical line)

The x-axis on the figure has been changed to reflect the sgRNA cuts sites and give location relative to them.

Response to Reviewers' comments

We would like to take the opportunity to thank the reviewers as well as the editor for their time and attention which have helped shape the manuscript. Please find below a point-by-point response to the latest comments.

Line 33: Can they be described here, maybe make space by reducing the explanation of NHEJ mutations etc. above?
Lines 34-5: This makes it sound like a mutation at the cut site didn't prevent homing for that sgRNA - needs rephrasing.
The abstract has been reworded to address these two comments.

Lines 67-70: it is not explicitly stated how a gene drive of this type biases its inheritance (through HDR)
Amended

Line 76: why non-sex specific?
This sentence has been clarified.

Line 78: Worth discussing here the large amount of variability in anopheles genomes and standing variation in target populations?
Some text relating to genetic variation and its implication in multiplexing has been added to the introduction.

Line 84: not sure dramatically - just means you need several events arising independently on the same genotype background
and second comment: it is dramatic - it is more or less the product of the individual probabilities, because once a drive conversion event happens the pre-existing r alleles are scooped out (rotavated **[redacted]**). But I agree, the way they have written is not clear and does not capture this subtelty.
This sentence has been modified.

Line 85: 'completely gene drive resistant'
This sentence has been modified.

Line 86: would move this sentence to line 73
Moved as suggested

Line 88: is this referring to gene drives in general or specifically to multiplexed gene drives?
This sentence has been reworded.

Line 88: There are two recent references on effects (or lack thereof) of sequence heterology in Anopheles that should be referenced - Tushar et al 2024 and Pescod et al. 2023
After examination of the suggested literature we have added some text here to include these suggested references.

Line 91: Slightly misleading wording - probably the same chance of mutations being functional, but the chance of all mutations in all gRNA sites being functional is low
This has been reworded.

Line 93: What was the first significant drawback? This section may need some restructuring for clarity

This section has been expanded to try to further explain how the two strategies differ.

Line 96: Worth mentioning that this would likely produce a non-functional protein, if the gRNA sites have been well designed?
Second comment: yes. There is a lot of potential misdirection going on here
Line 98: single gene or multiple genes?
Line 99: single construct could still target multiple sites without them being adjacent
This section has been substantially rewritten to address these comments.

Line 100: THIS could be a focal point for the paper i.e. if it were framed as experimentally testing this versus standard then it would have an increased level of novelty and interest.
We have tried to emphasize this point in a few key places in the abstract, introduction and discussion though we think the results with the classic multiplexing approach which has not had a great deal of empirical validation, are also important – we have tried to strike a balance between these two aspects.

Line 108: this is the totality of the genes in the ommochrome pathway? Some of the phrasing, and this is an example, is a bit clunky or imprecise throughout the introduction.
These sentences and some others in the introduction have been reworded to try to make them clearer.

Lines 110-111: Can you explain the eye phenotype seen in both hets and homs here
A description of the eye phenotype has been included in this section.

Line 114: two different strategies? be more explicit here
This sentence was already amended in the initial revision.

Line 126: in the cd gene
This sentence was already amended in the initial revision.

Line 132-134: I would have thought 136 nt was definitely close enough together to remove the entire chunk
Second review comment: Evidence from others of multiplexed gene drives with bigger gaps between the targets, or do they tend to all be this close together?
There are a handful of papers which have used multiple gRNAs and the spacing between them is very similar to what we have used here. Our previous work in Aedes aegypti used 4 gRNAs within 135bp (Anderson et al. 2024), work from Jackson Champer's group has used multiplexed gRNAs with regions of 114bp (Champer 2020, Chen 2026), and 92bp (Hou 2024) they also investigated two gRNAs with a spacing of 36bp. In Oberhofer et al 2018 and supported by further evidence in Champer et al 2020, they found that much more widely spaced gRNAs substantially reduced drive efficiency, as a result the consensus in the field is that the ideal for multiplexing is to use multiple gRNAs targeting closely spaced sequences. This sentence has been reworded.

Line 144: I don't understand this - homozygous mutants with what mutation?
Second reviewer comment: Having finished the paper - a 3bp deletion at the gRNA site that prevents homing (and produces a different eye phenotype? het vs hom phenotype? needs explaining)
Reviewer 1: is it not just a recessive mutation?
This section was already amended in the initial revision.

Lines 173-78: All of this needs a diagram to explain it
A diagram has been added to the supplemental information to explain meiotic drive.

Line 175: does this mean within the gene, causing a knockout?
No, genetic linkage merely indicates the genes are close together such that they are inherited together, as stated in the text the recombination rate is less than 1%, which means that 1% of the time these genes will separate through normal recombination.

Line 179: change term - or at least specify linked to the target locus (rather than linked to the drive allele)
This has been amended.

Line 188: this needs putting way further up, when the cd384 line is first mentioned
This information has been included at the first mention of the cd^{384R} *line.*

Line 189: Again could do with a diagram explaining the crosses, expected progeny and their eye phenotypes/fluorescent markers
This sentence has been modified as eye phenotype is not relevant in this particular experiment, a table has been added to the supplemental information which describes all the crosses and the expected genotype, phenotype and fluorescent markers.

Line 200: 'recognised by'
This has been amended.

Line 214-216: is there previous literature to be cited on this 'sweet spot'?
To our knowledge the additive multiplexing strategy has not been experimentally tested, with the only exploration being the theoretical mathematical modelling extensively cited. This theory paper does not explore the details of the molecular biology/design beyond the criteria that the recombination rate between the two targets sites should be low and that the two constructs should home independently.

Line 218: why not put these numbers on the figure panels?
We appreciate the suggestion, however we found including the value of the mean at the location of the point on the figure made it too busy, especially for those with very high homing rates where many of the individual data points are all clustered at the top of the graph.

Line 221: Is this a problem/what would be the significance for the experiment?
We believe this difference is due to the effectiveness of the sgRNA that targets this locus. It is quite common for different sgRNAs to have different inherent efficiencies. For this proof of principle experiment it makes no difference. For field deployment an sgRNA with higher efficiency would be more desirable.

Line 225: This is interesting - could this use case be touched on in the intro (the likelihood of residual gene drive resistance mutations existing after the gene drive has died out, and their potential to interact with other gene drives)?
This is the theoretical advantage of the additive multiplexing strategy, this has now been clearly detailed in the introduction.

Line 233: pretty sure this should say Fig 3B, right hand panel)
Yes, thank you, this has been amended.

Line 234: 'at the distal sgRNA target site'
This has been amended.

Line 239: on panel 5 there are not the same mean homing rate (with error) summary bars as there for the 225 transgene
The same mean homing rate and error bars are present in the figure, in the text it is explained that the mean homing rate was 99.59% ([95% CI] = [98.31-99.9]) the bars are simply too small to be seen within the scale of the figure.

Line 243: " at target site 225 or 384, respectively, in the cd gene"
This has been amended.

Line 248: why are these only given on panel B and not panel D?
The same mean homing rate and error bars are present in the figure, in the text it is explained that the mean homing rate was 99.59% ([95% CI] = [98.31-99.9]) the bars are simply too small to be seen within the scale of the figure.

Line 254: on what tissue was the sequencing performed? whole body or germline?
Whole body samples were collected, this is mentioned in the methods.

Line 254-255: To get such a high mutation rate I'm assuming only males with a mutated eye phenotype were collected?
We did not separate based on eye-phenotype; however, we do see mosaicism in the eyes of trans-heterozygotes expressing both Cas9 and the sgRNA likely due to zygotic expression and/or maternal deposition of Cas9, depending on the direction of the initial cross.

Line 265-267: was there any enrichment done by selecting for eye phenotype?
No, see above.

Line 280: i don't think that this is the case but this reads as though the remainder (32%) of reads that lacked all four target sites had multiple Cas9-induced SNPs or deletions that individually affect all of the four target sites. Was that the case?
Yes, that is the case, in the remaining reads we found two or more indels which individually disrupted all four sites.

Line 300: maybe I'm missing the point, but could they not have performed a few cage trials with these lines? particularly with the additive approach - put both gene drive lines in together and examine the proportion of individuals which have one/both constructs over generations
Second reviewer: I think a cage trial is not immediately necessary as it does point to a theoretical quirk that wouldn't be immediately obvious to all. They have published on this in the past, in Evol Apps I think, but this is at least experimentally going some way to address it.
As we have explored these multiplexing methods through mathematical models, and cage trials are a substantial amount of work when done correctly, we sought to model a cage-trial deriving parameters for these strains. This allows us to investigate the performance without the significant investment in a cage trial.

Line 311: not realistic for pop supp strategy where target gene is haplosufficient (to a point)
Second reviewer: There is a wider point that they should be clearer about - people will mistakenly conflate these ideas for all types of gene drives but is obviously most relevant where target sites (and resulting repaired alleles) are mostly neutral. E.g. see line 408/409
The fitness costs estimated here are solely due to carrying a transgene and are not intended to be reflective of a population suppression system. In the first paragraph of this section we state that we

are modelling a population modification drive. In the prior sentence we explain that we are modelling a gene drive in a neutral site with only a moderate fitness cost.

Line 312-313: what is the starting assumption for the additive approach - that the two modules are linked? or that they're separately circulating? If separate, either initially or subsequently, what happens when they meet across homologues. Presumably they go into linkage but the effect, and outcomes from, of potential cleavage of both chromosomes could be tested experimentally.
The introduction has been extended to discuss the idea behind the additive strategy a bit more. In principle such elements could be released separately or together, as the reviewer notes. In the instance cited, for the purpose of the modelling they are together in the initial release generation, this allows a 'fair' comparison to the classical multiplex strategy which has four sgRNAs which are (obviously) released together. Experimental exploration of this is beyond the scope of the current work, but this is modelled extensively in a previous publication, Edgington et al 2020.

Line 315: as in a single gene? or a single section of the chromosome?
It could be either a single gene or two different genes, as long as they are sufficiently close to eliminate recombination, thus we used the more general phrase "genomic region".

Line 407-8: As above, need to be clearer about the scenarios - in terms of different drive elements and intended fitness costs (e.g. popn suppression drives when in homozygosity) versus unintended fitness costs. The dynamics of the probability of generating resistant mutations that would be selected are different.
In the previous revision we have expanded our discussion to include how population modification and suppression drives differ in regards to fitness costs and selection of resistance alleles.

Line 411: Is this comparable - e.g. were these rates for mutations in an essential gene?
This is comparable, the cited study was targeting the neutral ommochrome pathway gene kmo.

Line 414-5: was it not under zpg promotion?
Yes, it was, but we did observe zygotic expression and somatic cutting with this Cas9 line.

Line 447: If they're both targeting the same gene this is unlikely though isn't it?
Second reviewer: maybe that just means it needs to be stated clearly here - that additive multiplexing may not work if multiple genes are targeted, as that would allow for these cumulative fitness reduction effects
This has been clarified in the text.

Line 456-7: Surely the most important point with this will be choosing the correct target gene?
This statement has been further expanded.

Response to Reviewer’s comments

We would like to thank the reviewer’s for their time in considering our manuscript. Below is a point by point response to the remaining comments.

REVIEWERS' COMMENTS

Reviewer #1 (Remarks to the Author):

I am happy with the reviewers' replies to comments.

I believe Figure 5 should say "each sgRNA target site" not "each sgRNA"

This has been corrected.

Reviewer #2 (Remarks to the Author):

I co-reviewed this manuscript with one of the reviewers who provided the listed reports. This is part of the Nature Communications initiative to facilitate training in peer review and to provide appropriate recognition for Early Career Researchers who co-review manuscripts.
